# Klotho protects the osteogenic function of human periodontal ligament stem cells in periodontitis by inhibiting NOX4-mediated ferroptosis

**DOI:** 10.1186/s13287-026-04894-w

**Published:** 2026-01-16

**Authors:** Chuanmiao Lin, Junbin Wei, Tingting Zhao, Lingling Chen, Shuxuan Rong, Junkun Zhan, Wenjia Lai, Yan Wang, Yunyi Xie, Huan Chen

**Affiliations:** 1 Hospital of Stomatology, Guanghua School of Stomatology, Guangdong Provincial Key Laboratory of Stomatology, Sun Yat-Sen University, Guangzhou, 510055 China; 2 School of Stomatology, Clinical Research Platform for Interdiscipline of Stomatology, The First Affiliated Hospital of Jinan University, Jinan University, Guangzhou, 510630 China

**Keywords:** Klotho, Ferroptosis, Periodontitis, Osteogensis, NOX4, Human periodontal ligament stem cells

## Abstract

**Background:**

Periodontitis, the leading cause of tooth loss worldwide, is closely linked to the compromised regenerative capacity of human periodontal ligament stem cells (hPDLSCs). Stem cell-based tissue engineering is a promising treatment for periodontitis. Sustaining the osteogenic potential of hPDLSCs against adverse conditions following transplantation is critical for successful periodontal tissue engineering. Recent research increasingly underscores ferroptosis as a crucial target for periodontitis treatment, whereas reactive oxygen species (ROS) contribute to ferroptosis initiation and progression. Klotho, an anti-aging protein, has been shown to protect hPDLSC osteogenic function under oxidative stress in our previous study. However, whether Klotho can provide protection against ferroptosis and maintain osteogenic function of hPDLSCs in an inflammatory environment remains elusive.

**Methods:**

Ferroptosis level and the expression of Klotho in hPDLSCs under normal and inflammatory conditions were compared via single-cell RNA sequencing and validation experiments. Stable Klotho-overexpressing hPDLSCs (hPDLSCs-ov-KL) cell line was established and the impact of Klotho on ferroptosis was assayed. Subsequently, the effect of Klotho overexpression on hPDLSC osteogenesis was evaluated under in vitro inflammatory environment and in vivo periodontitis model of C57BL/6 mice. Additionally, the underlying molecular mechanism of Klotho effect on hPDLSCs under the inflammatory environment was investigated.

**Results:**

Ferroptosis was activated and the expression of Klotho was reduced in hPDLSCs under LPS-stimulated inflammatory environment, consistent with the results in hPDLSCs of periodontitis via single-cell RNA sequencing. Further experiments confirmed Klotho overexpression effectively suppressed ferroptosis in hPDLSCs and markedly preserved the hPDLSC osteogenic capacity under in vitro inflammatory environment. In vivo, injection of hPDLSCs-ov-KL could effectively promote periodontal tissue repair in the mouse model of periodontitis. From the perspective of molecular mechanism, Klotho notably inhibited NOX4 expression in hPDLSCs under the inflammatory environment and NOX4 overexpression in hPDLSCs-ov-KL significantly increased intracellular ferroptosis, leading to compromised Klotho protective effect.

**Conclusion:**

Our study highlighted the significant protective effect of Klotho on counteracting hPDLSC ferroptosis via the inhibition of NOX4 expression, therefore restoring the impaired osteogenic function of hPDLSCs in both in vitro inflammatory environment and in vivo periodontitis model, which might provide a promising strategy for periodontal tissue regeneration engineering.

**Graphical Abstract:**

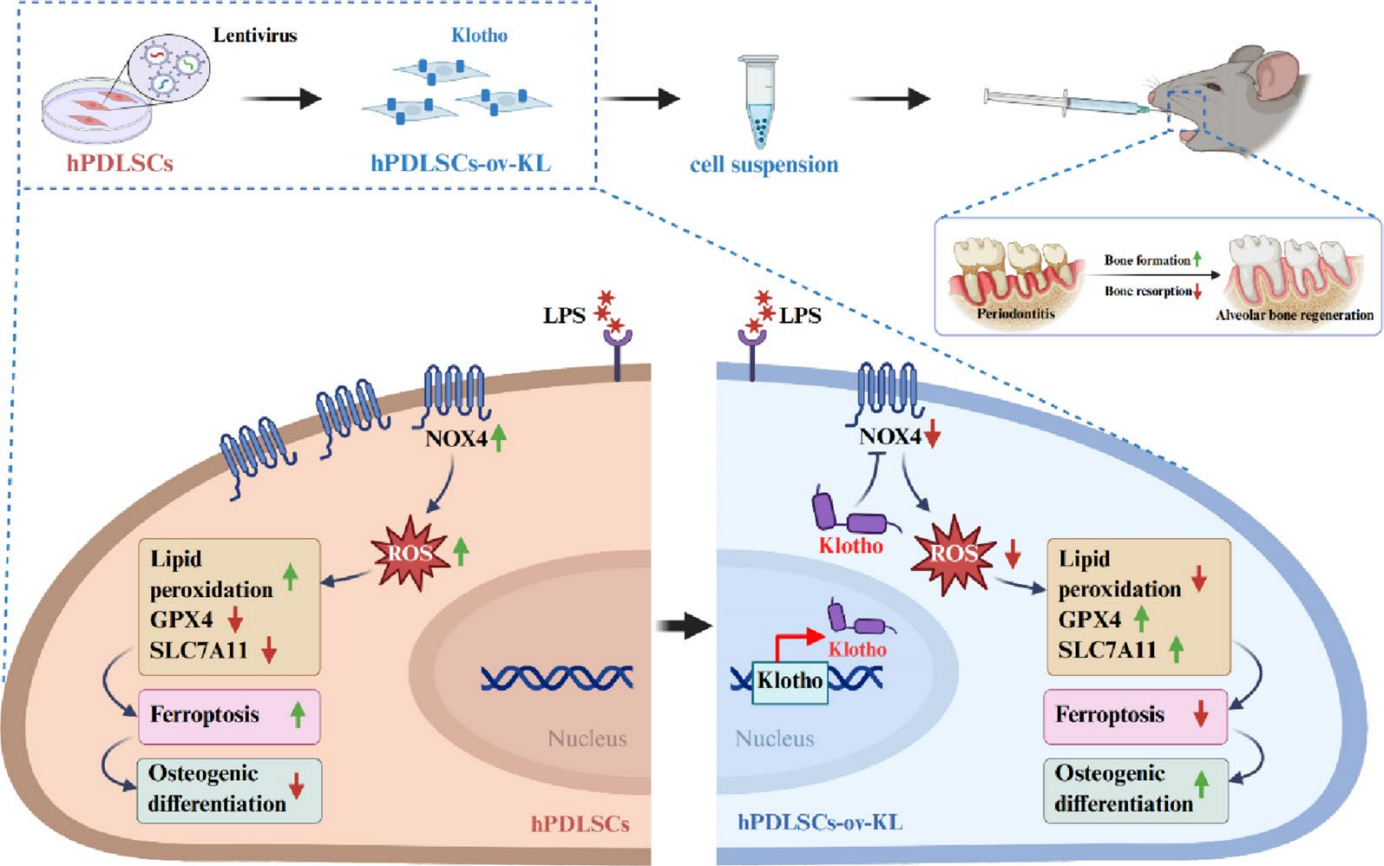

**Supplementary Information:**

The online version contains supplementary material available at 10.1186/s13287-026-04894-w.

## Background

Periodontitis is a chronic inflammatory disease affecting tooth-supporting tissues, and it has resulted in tooth loss and reduced quality of life in 45%–50% of adults globally [1, 2]. Treatment focuses on regenerating periodontal tissues, especially alveolar bone [3]. Human periodontal ligament stem cells (hPDLSCs) with strong self-renewal, multi-directional differentiation, and immune-regulatory abilities, are ideal for periodontal tissue engineering [4]. However, their function and stemness decline in adverse microenvironment such as inflammation, oxidative stress, and hypoxia after transplantation [5]. Therefore, developing novel strategies to enhance hPDLSC resilience against inflammatory stimuli and maintain osteogenic potential are crucial for effective periodontitis therapy [6].

Ferroptosis is a type of cell death caused by disrupted iron metabolism and redox homeostasis, resulting in mitochondrial shrinkage, glutathione (GSH) depletion, and excessive lipid peroxidation [7]. Recent studies show that ferroptosis plays a key role in periodontitis, where iron overload and excess reactive oxygen species (ROS) accumulation worsen alveolar bone loss in mice [8, 9]. Ferroptosis is closely associated with bone homeostasis, contributing to mesenchymal stem cell death in infected bone microenvironment [10]. In periodontitis, hPDLSCs show increased sensitivity to ferroptosis, with upregulated oxidative pathway and decreased expression of SLC7A11 and GPX4, and ferroptotic osteoblast lineage cells and hPDLSCs directly disrupt bone homeostasis [11, 12]. These findings suggest that protecting hPDLSCs from ferroptosis may be a promising therapeutic strategy for promoting bone regeneration in periodontitis, and the underlying mechanism within ferroptosis and inflammatory hPDLSCs needs to be clarified.

Cellular ROS, primarily regulated by mitochondria, drive oxidative stress [13]. Excess ROS damage lipids, particularly polyunsaturated fatty acids in cell membranes, resulting in lipid peroxidation and cellular dysfunction [14]. Iron exacerbates ROS through Fenton reactions. When antioxidant defenses such as GSH and GPX4 are compromised, lipid peroxides accumulate, triggering ferroptosis [15, 16]. Thus, ROS plays a key role in initiating and advancing ferroptosis, thereby amplifying cellular damage and dysfunction.

*Klotho*, an anti-aging gene, discovered by Japanese scientist Kuro-o in 1997 [17], is known for its antioxidant properties. Our previous research showed that Klotho reduces ROS and preserves the osteogenic function of hPDLSCs under oxidative stress [18]. Recent study also observes marked decrease in Klotho levels and increase in ferroptosis in aging renal tissue and cells following irradiation, indicating an inverse relationship between Klotho and ferroptosis [19]. Meanwhile, studies have highlighted a link between Klotho and periodontal disease. *Klotho* gene knockout mice show abnormal alterations in periodontal histomorphology [20]. Furthermore, lower serum Klotho levels are associated with more severe periodontitis, suggesting its potential as a biomarker for disease progression [21, 22]. These results prompted us to consider whether Klotho expression could enhance hPDLSC biogenesis to regulate cellular ferroptosis in inflammatory status.

Klotho plays a crucial role in regulating the mitochondrial function and enzymatic system [18, 23]. NADPH oxidase 4 (NOX4) is an enzyme that continuously generates ROS without external stimuli [24]. Emerging evidence underscores its role in inducing ferroptosis and disrupting bone metabolism [25–27]. NOX4 exacerbates pathological bone resorption by driving ROS-dependent osteoclast differentiation [28]. Notably, Zhang et al. showed that NOX4-mediated ferroptosis in osteoblasts resulted in osteoporotic bone loss caused by iron overload [29]. Additionally, NOX4 expression in Klotho-depleted podocytes in diabetes was increased [30], indicating that Klotho may regulate cellular biogenesis via NOX4. However, whether NOX4 involves in the osteogenic function of inflammatory hPDLSCs and its interaction with Klotho remain to be elucidated.

In this study, we aimed to explore the effects and mechanism of Klotho on counteracting hPDLSC ferroptosis and preserving hPDLSC osteogenic function under the inflammatory environment. We have confirmed that Klotho inhibited NOX4-mediated ferroptosis, therefore restoring the impaired osteogenic function of hPDLSCs in both in vitro inflammatory environment and in vivo periodontitis model of C57BL/6 mice. This reveals a promising therapeutic pathway that links ferroptosis, oxidative stress, osteogenesis and inflammation, thus offering potential for treating inflammatory bone diseases, including periodontitis.

## Methods

### Bioinformatic analysis of single-cell RNA sequencing

Single-cell RNA sequencing data were obtained from the GEO database, numbered GSE171213 [31]. This dataset is a public database and includes periodontal ligament tissues from four healthy people and five patients with periodontitis. The data after downloading were read and further analyzed using Seurat (v4.3.1). Violin, dot and feature plots were used to visualize the marker gene expression to confirm cluster identity. We focused on the clusters with increased CytoTRACE2 scores and the expression of typical mesenchymal stem cell (MSC) markers, including CXCL12 and PDGFRA, to identify possible MSCs [32]. The ferroptosis-associated gene set was obtained from the MsigDB database. The score of the ferroptosis gene set was calculated using the AddModuleScore function after screening the MSCs using CytoTRACE2.

### Analysis of the microarray data

Microarray data were obtained from GSE16134. Furthermore, for each PD sample, GSVA was utilized to calculate the ferroptosis functional enrichment score, and the Spearman correlation analysis was used to determine the correlation between *Klotho* gene expression and ferroptosis.

### Cell culture and treatment

The hPDLSCs were isolated and cultured as previously described [18]. Periodontal ligament tissues were aseptically harvested from the third molars of 12 healthy donors (18–25 years, 6 men and 6 women) after obtaining approval from the Ethics Committee of the Affiliated Stomatological Hospital of Sun Yat-sen University (KQEC-2022-116-01). All donors provided written informed consent prior to tissue collection. The isolated cells were expanded and cultured in α-minimum essential medium (α-MEM; Gibco, USA) supplemented with 10% fetal bovine serum (FBS; Gibco, USA) and 100 units/mL streptomycin/penicillin (HyClone, USA) at 37 °C in 5% carbon dioxide. Cells from passages 3–5 were used for subsequent experiments. To simulate inflammatory conditions, the hPDLSCs were exposed to 4 µg/mL of LPS (Sigma, USA) in their respective media for 48 h.

### Establishment of Klotho-overexpressing hPDLSCs

Primers were designed based on the target gene sequence from GenBank (NM_004795.4) of α-Klotho ( Forward : 5′-CTAGGCGCCGGAATTAGATCTCCTGGCTCCCGCGCAGCAT.

G-3′; Reverse : 5′-GTAGAATTCGTTAACCTCGAGCTATTTGTAACTTCTTCTGCCTT-.

3′ ). The plasmids were purchased from the Addgene website (Catalog #1771) and utilized as the templates for polymerase chain reaction (PCR) amplification. The Klotho plasmids were digested with BglⅡ to generate linear templates. The PCR-amplified Klotho fragments and linearized vector were recombined using the Exnase II Cloning Kit (Vazyme, China). The recombinant plasmids were transformed into *Escherichia coli* DH5α, purified using a plasmid isolation kit (Omega, USA), and sequenced for validation. Plasmids with correct Klotho overexpression sequences and empty ones used as a control were named pMSCV-ov-KL and pMSCV-ov-control, respectively. For lentivirus production, 293T cells were transfected with 7.5 µg of pMSCV-ov-KL or pMSCV-ov-control alongside the PIK packaging vector. Furthermore, viral supernatants were harvested at 48 h post-transfection and utilized to infect the hPDLSCs for 6 h. Puromycin selection was performed at 48 h post-infection. Successful infection was confirmed using RT-qPCR and Western blotting. Klotho-overexpressing hPDLSCs were named as hPDLSCs-ov-KL (abbreviated as Ov-KL in figures), and empty vector control hPDLSCs were named as hPDLSCs-ov-con (abbreviated as Ov-Con in figures). Normal hPDLSCs abbreviated Con in figures.

### Quantitative reverse transcription polymerase chain reaction (RT-qPCR)

Klotho expression levels, ferroptosis-associated markers, and osteogenic markers were measured by RT-qPCR. Total RNA was extracted from the cells using the Ultrapure RNA kit (CWBIO, China), and mRNA was reverse transcribed into cDNA using HiScript^®^ III RT SuperMix (Vazyme, China), according to the manufacturer’s instructions. A Light Cycler System with the RealStar Fast SYBR RT-qPCR Mix (Genstar, China) was used to conduct RT-qPCR. Primer sequences used for target gene amplification were listed in Table S1. Glyceraldehyde-3-phosphate dehydrogenase (GAPDH) was served as the internal control. The cycling conditions were as follows: incubation at 95℃ for 5 min, 40 cycles of denaturation at 95℃ for 15 s, annealing at 60℃ for 20 s, and extension at 72℃ for 20 s. The 2^−ΔΔCt^ method was used to calculate relative gene expression.

### Western blot analysis

Klotho expression level, ferroptosis-related markers, and osteogenic markers were measured via Western blotting. The cells were harvested and lysed on ice in radioimmunoprecipitation assay buffer (Beyotime, China) for 30 min. The total protein concentrations were measured via a Pierce bicinchonic acid protein assay kit (Thermo Scientific, USA). Furthermore, 30 µg of the protein was separated using 10% sodium dodecyl sulfate-polyacrylamide gel (SDS-PAGE) and transferred into nitrocellulose (NC) membranes. Subsequently, the membranes were blocked with 5% (w/v) nonfat milk for 1 h at room temperature and incubated overnight at 4 °C with primary antibodies. After washing, the membrane was incubated with secondary antibodies (1:5000, Abmart). Protein bands were visualized and documented using the the ChemiDoc Imaging System (Biorad, USA). The primary antibodies were used: Klotho (1:1000, Proteintech), GPX4 (1:2000, Abmart), SLC7A11 (1:1000, Abmart), ALP (1:1000, Abmart), RUNX2 (1:1000, Boster), NOX4 (1:2000, HuaBio), GAPDH (1:50000, Proteintech) and β-actin (1:1000, PTM BIO).

### In vitro osteogenesis assays

hPDLSCs were seeded in 24-well plates at a 2 × 10^⁴^ /mL density and cultured until they reached 70%–80% confluence. Cells were subsequently induced with osteogenic differentiation medium (α-MEM supplemented with 10% FBS, 10 mM β-glycerophosphate, 10 nM dexamethasone, and 50 µg/mL ascorbic acid). After 7 days of osteogenic induction, osteogenic markers were measured by RT-qPCR and Western blotting. Alkaline phosphatase (ALP) staining and the ALP activity were measured according to the manufacturer’s protocol of BCIP/NBT Alkaline Phosphatase Color Development Kit (Beyotime, China) and the Alkaline Phosphatase Assay Kit (Beyotime, China), respectively. At 21 days after osteogenic induction, calcium deposits were detected using Alizarin Red staining (ARS) after induction. The cells were fixed with 4% PFA for 20 min, and the stained calcium deposits were observed and photographed under an inverted microscope (Olympus, Japan). Semi-quantitative analysis involved dissolving calcium nodules with 10% cetylpyridinium chloride (Yuanye, China) and recording absorbance at 562 nm using a microplate reader (BioTek, USA).

### Malondialdehyde (MDA) and Fe^2+^ detection under in vitro conditions

MDA levels were quantified using a Lipid Peroxidation MDA Assay Kit (Beyotime, China) following the manufacturer’s instructions. Cells were harvested and lysed in lysis buffer to obtain the supernatant. A freshly prepared TBA-containing working solution was added to the supernatant, which was subsequently incubated at 100 °C for 15 min. The absorbance at 532 nm was recorded.

FerroOrange (DoJINDo, Japan) was used according to the manufacturer’s protocol to detect intracellular Fe^2+^. Cells were treated with 1 µmol/L FerroOrange with basal culture medium at 37℃ for 30 min. The cells were subsequently detected under a confocal laser scanning microscopy (Zeiss, Germany).

### Transmission electron microscopy (TEM)

The mitochondrial morphology of the cells was observed using the TEM (Hitachi, Japan). Cells were fixed in 2.5% glutaraldehyde and subsequently embedded in epoxy resin. Ultrathin sections were treated with uranyl acetate and lead citrate for staining before being analyzed.

### ROS measurement

Intracellular ROS levels were evaluated using the ROS Assay Kit (Beyotime, China). The cells were stimulated and incubated following the manufacturer’s instructions. After washing, the cells were observed under a confocal laser scanning microscope (Zeiss, Germany).

### Animal experiments

All animal experiments were approved by the Institutional Animal Care and Use Committee, Sun Yat-Sen University (SYSU**-**IACUC**-**2024**-**001177) and conducted in accordance with the national guidelines for animal welfare. The study is reported in line with the ARRIVE guidelines 2.0 (Supplementary Material 3). Thirty male C57BL/6 mice (6–8 weeks old) were obtained from the Guangdong Medical Laboratory Animal Center (Guangzhou, China). The animals were housed under specific pathogen**-**free (SPF) conditions in a controlled environment. The sample size of six mice per group was determined to be sufficient for detecting statistical significance based on pre-experimental data. Six mice were randomly assigned to the healthy control Normal group. Periodontitis was induced in the remaining 24 mice under general anesthesia using 1% pentobarbital sodium by placing a silk ligature around the maxillary second molars. Ligatures were checked every two days. After two weeks, the ligated mice were randomly divided into four experimental groups (*n* = 6 per group). The groups were: PD + PBS: periodontitis model + local injection of PBS; PD + Con: periodontitis model + local injection of hPDLSCs; PD + Ov**-**Con: periodontitis model + local injection of hPDLSCs**-**ov**-**con; PD + Ov**-**KL: periodontitis model + local injection of hPDLSCs**-**ov**-**KL.

The experimental unit was the individual mouse. For cell treatments, 1 × 10⁵ cells in 50 µL PBS were injected using a microinjector (Gaoge, China) beneath the proximal and distal mucosa on the palatal side of the second molar, three times per week throughout the 28**-**day experimental timeline. Animals were monitored every three days for signs of significant weight loss, impaired food intake, or aberrant behaviors. The study would be terminated if any of these predefined humane endpoint was met. At the terminal stage of the experiment, mice were euthanized by an intraperitoneal overdose of 1% pentobarbital sodium following the AVMA Guidelines for the Euthanasia of Animals (2020 edition). Maxillary bones were collected for Micro-CT and histological analysis. To minimize bias, group assignment and administration of treatments were performed by researchers CML and JBW, while outcome assessment and statistical analysis were performed by personnel blinded to the group allocations.

### Micro-computed tomography examination (Micro-CT)

Micro-CT scanner (SCANCO, Switzerland) was used for scanning the fixed maxillary samples. Mimics (Mimics 21.0, Belgium) was used for generating and scanning three-dimensional reconstructions. Measurements were taken from the reconstructed maxilla, focusing on the distances from the proximal-medial, intermediate-medial, and distal-medial cementoenamel junctions to the highest point of the alveolar bone crest of the second molar. Six points were measured on the buccal and palatal sides, and the average values were calculated for statistical analysis.

### Histological and immunohistochemistry staining

Maxillary specimens were decalcified in 10% EDTA, dehydrated via an ethanol series, and paraffin-embedded. Serial 4 μm sections via defect sites underwent histological staining with hematoxylin and eosin (H&E) or Masson’s trichrome (Servicebio, China). Osteoclast quantification involved TRAP staining (Servicebio kit), with multinucleated TRAP-positive cells defined as active osteoclasts. Immunohistochemistry for bone sialoprotein (BSP) expression was conducted, and all sections were digitally imaged using a slide scanner (Leica, Germany).

### Transient overexpression and Inhibition of NOX4

Empty plasmid and NOX4 plasmid were purchased from Miaoling Plasmid Platform (Wuhan, China). Subsequently, they transformed into *Escherichia coli* DH5α for amplification. We performed endotoxin-free extraction of the plasmids after confirming successful sequencing. These were then transfected into hPDLSCs**-**ov**-**KL using a transfection kit (Hanbio, China). hPDLSCs-ov-KL overexpressing NOX4 were abbreviated as Ov**-**KL + Ov**-**NOX4 in figures, and empty vector control hPDLSCs**-**ov**-**KL were abbreviated as Ov**-**KL + Ov**-**CTR in figures. The overexpression efficiency was evaluated using RT-qPCR and Western blotting. We obtained the NOX4 inhibitor GLX351322 (MCE, USA) to inhibit NOX4 expression in the hPDLSCs-ov-KL.

### Statistical analysis

Statistical analysis was conducted using GraphPad Prism 9.4. The data were presented as mean ± standard deviation (SD). To determine differences between the two groups, an unpaired t-test was used, and for multiple groups, a one-way ANOVA was applied. *P* values less than 0.05 indicated statistical significance.

## Results

### Bioinformatic analysis revealed differential gene expression of Klotho and ferroptosis pathway in the hPDLSCs of patients with periodontitis

**Fig. 1:**
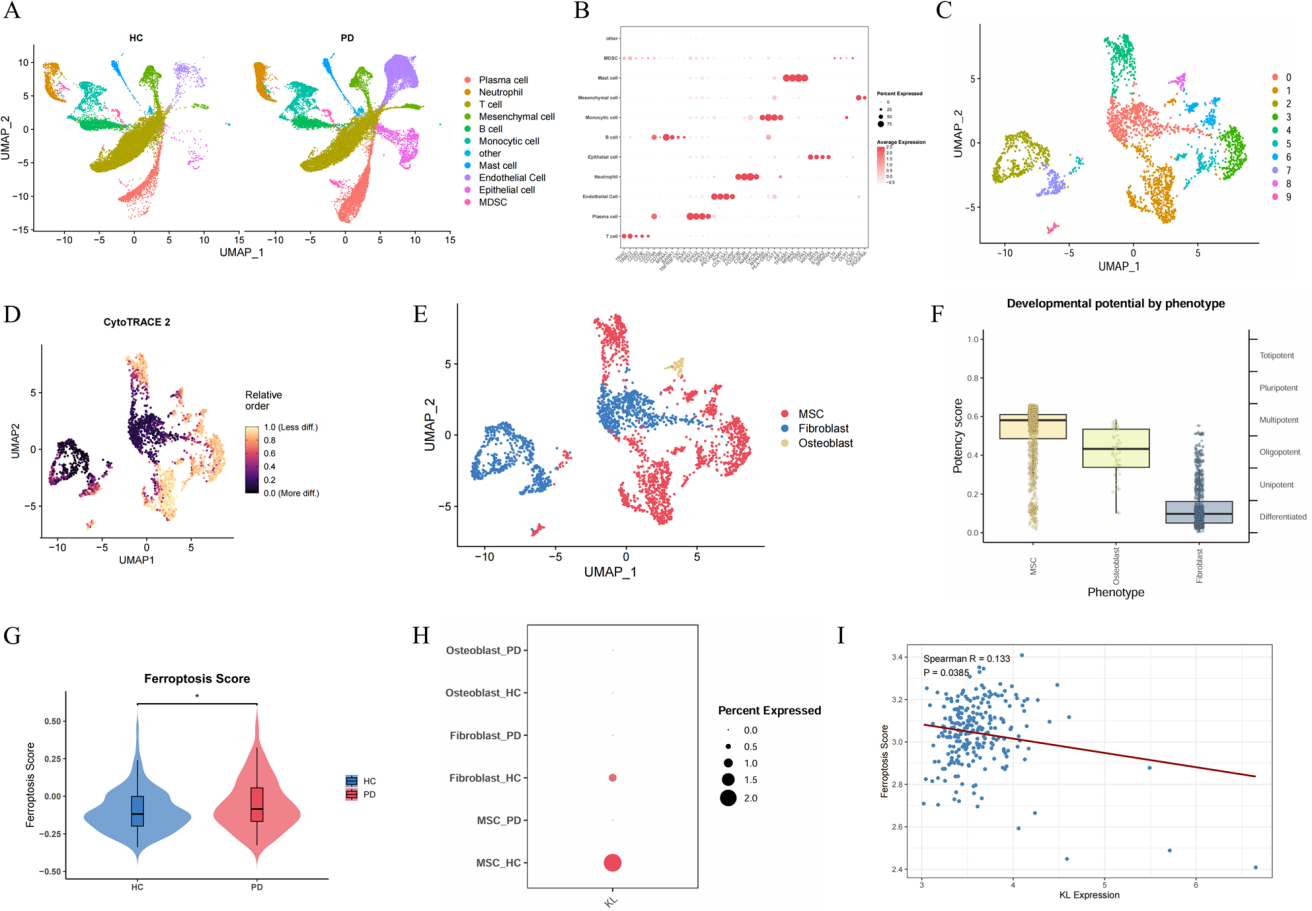
Single-cell sequencing showed the activation of the ferroptosis pathway in the MSCs of patients with periodontitis. **A** UMAP plot revealed periodontal ligament cell subpopulations in healthy patients and patients with periodontitis. **B** Bubble plot revealed the markers of cell subpopulations. **C** UMAP plot illustrated the mesenchymal cell subpopulations. **D** UMAP visualized the potential of the mesenchymal cell subpopulations. **E** UMAP revealed the classification annotation of the mesenchymal cell subpopulations. **F** Box plot illustrated the MSCs’ differentiation potential. **G** Violin plot illustrated the MSCs’ ferroptosis score. **H** Bubble plot showed the *Klotho* gene expression in the mesenchymal cell subpopulations. **I** Correlation between Klotho expression and ferroptosis score of the patients with periodontitis. (*P < 0.05)

Single-cell transcriptomic data from GEO database profiling of human periodontal ligament tissues from healthy donors (*n* = 4) and patients with periodontitis (*n* = 5) (Fig. S1A, B) identified 15 initial clusters post dimensionality reduction (Fig. S1C). Definitive cell annotation identified 11 lineages: T cells (CD3D/CD3E), B cells (CD79A/CD79B), plasma cells (IGHG1/IGHG2), endothelial cells (PECAM1/COL15A1), neutrophils (FCGR3B/CSF3R), monocytes (MS4A6A/CST3), mast cells (TPSAB1/MS4A2), epithelial cells (KRT6A/KRT5), myeloid-derived suppressor cells (LTF/CAMP), and mesenchymal cells (CXCL12/PDGFRA), with marker specificity validated via expression bubble plots and cluster-resolution distributions (Fig. S1D). Finally, the cells were classified into 11 subpopulations (Fig. 1A). Furthermore, the cell markers were visualized using bubble plots (Fig. 1B). CXCL12^+^PDGFRA^+^ mesenchymal cells were isolated from periodontal ligament subpopulations (Fig. 1C) and subclustered into 10 distinct groups. CytoTRACE2 analysis identified cluster 1,3,4,5,6 and 9 as multipotent MSCs based on the stemness scores (Fig. 1D–F, Fig. S1E). The extracted MSCs’ ferroptosis pathway was scored, and the results revealed that the MSCs’ ferroptosis score in patients with periodontitis patients was substantially upregulated (Fig. 1G), and ferroptosis-related genes were also markedly activated in them (Fig. S1F). This indicated that the ferroptosis process is activated in the hPDLSCs of patients with periodontitis. We further explored the *Klotho* gene expression in the MSCs. The expression of the *Klotho* gene in the hPDLSCs of patients with periodontitis was markedly downregulated (Fig. 1H). Additionally, the *Klotho* gene expression was negatively correlated with ferroptosis by analyzing the tissue samples from patients with periodontitis in microarray data. This indicated that the activation of the ferroptosis pathway in the hPDLSCs is associated with Klotho gene downregulation (Fig. 1I).

### Ferroptosis hindered the osteogenic function of hPDLSCs under the inflammatory environment

**Fig. 2:**
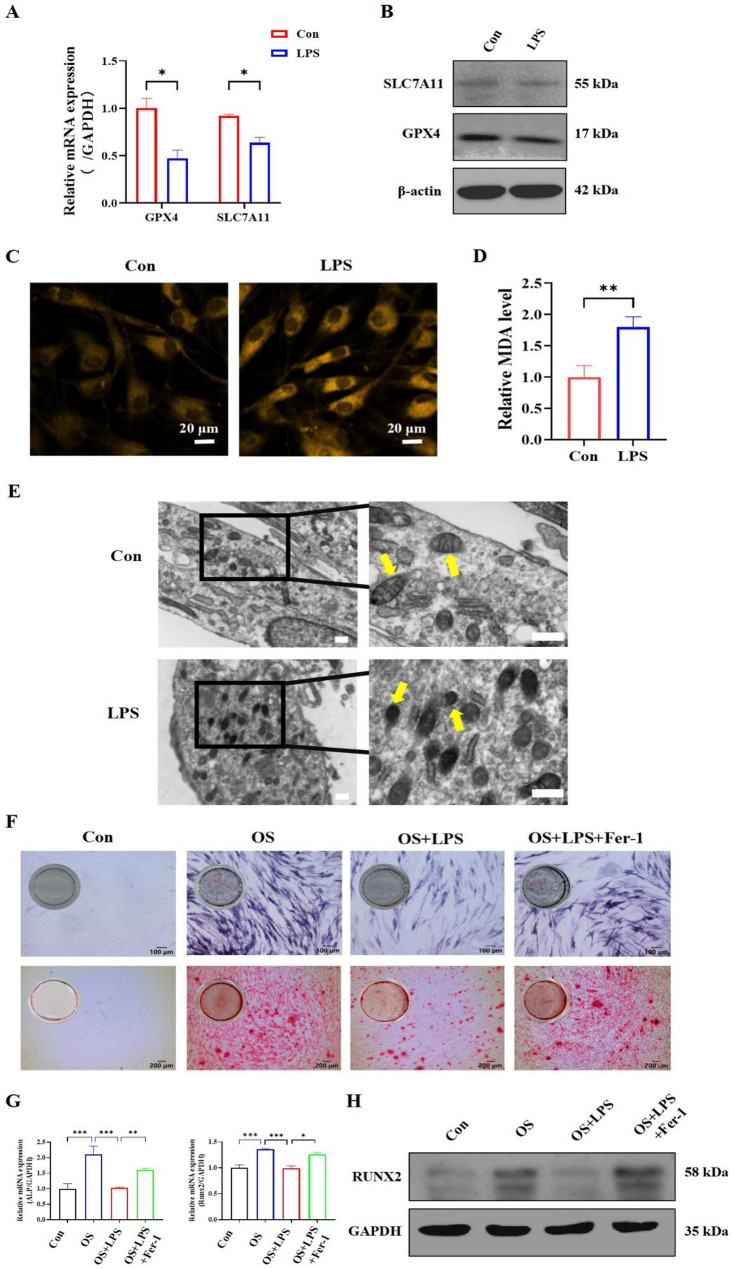
Ferroptosis level and osteogenic capacity of hPDLSCs under the inflammatory environment. **A** The mRNA expression levels of the ferroptosis-associated genes using RT-qPCR. **B** The protein expression levels of GPX4 and SLC7A11 by Western blotting. Uncropped images were provided in Supplementary material 2: Original Images for Western blots. **C** Measurement of the intracellular Fe^2+^ levels using a FerroOrange fluorescent probe (bar = 20 μm). **D** Measurement of lipid peroxidation by MDA assay. **E** TEM images of the mitochondrial structure (bar = 0.5 μm). **F** ALP staining of each group (bar = 100 μm). ARS of each group (bar = 200 μm). **G**, **H** The mRNA and protein expression levels of the *ALP* and *Runx2* genes of each group via RT-qPCR and Western blotting. Uncropped images were provided in Supplementary material 2: Original Images for Western blots. (*P < 0.05, **P < 0.01, ***P < 0.001, *n* = 3)

Cells isolated from human periodontal ligament tissue fulfilled the minimal criteria for MSCs and were identified as hPDLSCs. (Fig. S2). We conducted the assessments associated with the interplay between ferroptosis and inflammatory hPDLSCs. To establish an in vitro inflammatory model, gradient concentrations of LPS treating hPDLSCs was tested and 4 µg/mL of LPS for 48 h was selected to simulate inflammatory environment in this study (Fig. S3). Under the inflammatory environment, RT-qPCR and Western blotting showed that GPX4 and SLC7A11, the antioxidant defenses to ferroptosis, were markedly reduced in hPDLSCs (Fig. 2A and B), suggesting the occurrence of ferroptosis. FerroOrange fluorescent probe revealed a substantial increase of intracellular Fe²⁺ under the inflammatory environment (Fig. 2C). MDA, the indicator of lipid peroxidation, was significantly elevated in inflammatory hPDLSCs compared to normal control (Fig. 2D). Additionally, TEM showed shrunken mitochondria with degraded cristae, characteristic of ferroptosis (Fig. 2E). Collectively, these ferroptosis-related indicators confirmed the occurrence of ferroptosis in hPDLSCs exposed to inflammatory stimuli. Meanwhile, we investigated the impact of ferroptosis occurrence on the osteogenic function of hPDLSCs under the inflammatory environment. We observed that inflammation not only induced ferroptosis in hPDLSCs but also concurrently impaired their osteogenic function. However, this impaired function was restored following treatment with a ferroptosis inhibitor (Fig. 2F, G, and H). This underscores the role of ferroptosis in influencing the osteogenic function of hPDLSCs during inflammation.

### Klotho overexpression inhibited the ferroptosis of hPDLSCs under the inflammatory environment

**Fig. 3:**
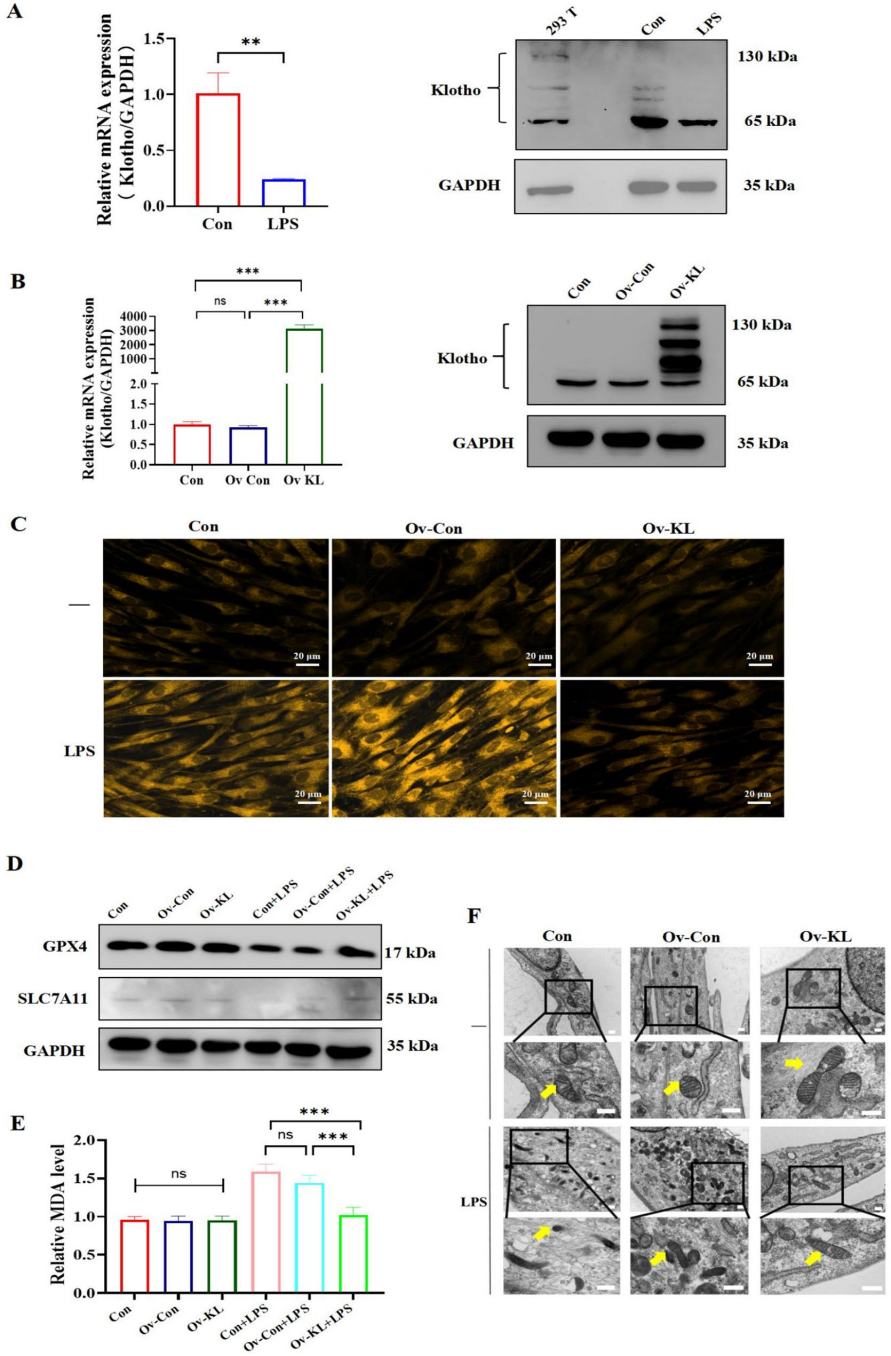
Klotho overexpression effectively resisted ferroptosis in hPDLSCs under the inflammatory environment. **A** The mRNA and protein expression levels of the *Klotho* genes in hPDLSCs under normal and LPS conditions by RT-qPCR and Western blotting. Lysates from 293T cells served as a positive control for the Klotho antibody. Uncropped images were provided in Supplementary material 2: Original Images for Western blots. **B** The mRNA and protein expression levels of *Klotho* genes after Klotho overexpression in hPDLSCs via RT-qPCR and Western blotting. Uncropped images were provided in Supplementary material 2: Original Images for Western blots. **C** Measurement of the intracellular Fe^2+^ levels using a FerroOrange fluorescent probe (bar = 20 μm). **D** Measurement of the protein expression levels of GPX4 and SLC7A11 via Western blotting. Uncropped images were provided in Supplementary material 2: Original Images for Western blots. **E** Detection of the lipid peroxides levels in each group via the MDA assay. **F** TEM images of the mitochondrial structure (bar = 0.5 μm). (**P < 0.01, ***P < 0.001, ns: not significant, *n* = 3)

Bioinformatic analysis in Fig. 1H showed the decreased expression of Klotho in hPDLSCs of periodontitis. Further, we assessed the change of Klotho expression in hPDLSCs under the inflammatory environment. RT-qPCR and Western blotting showed that inflammatory environment significantly attenuated Klotho expression levels in hPDLSCs (Fig. 3A). Considering this and the bioinformatic analysis result in Fig. 1I, Klotho was speculated to be associated with the ferroptosis of hPDLSCs. We tested this hypothesis by establishing hPDLSCs that overexpressing Klotho stably. RT-qPCR and Western blotting showed that hPDLSCs-ov-KL highly overexpressed the Klotho mRNA and protein (Fig. 3B). We next examined the effect of Klotho overexpression on ferroptosis in hPDLSCs under inflammatory environment. FerroOrange staining showed that Klotho overexpression attenuated the inflammation-induced increase in Fe^²⁺^ levels (Fig. 3C). Western blotting demonstrated that Klotho overexpression rescued the decreased expression of the anti-ferroptosis proteins GPX4 and SLC7A11 (Fig. 3D). MDA assays revealed that Klotho overexpression reduced the elevated lipid peroxidation levels (Fig. 3E). Furthermore, mitochondrial TEM showed clearer mitochondrial ultrastructure, outer membrane and cristae in the hPDLSCs-ov-KL group under inflammation compared to the control groups. (Fig. 3F). Hence, Klotho overexpression resisted the ferroptosis of hPDLSCs under the inflammatory environment.

### Klotho overexpression sustained hPDLSC osteogenesis under the inflammatory environment

**Fig. 4:**
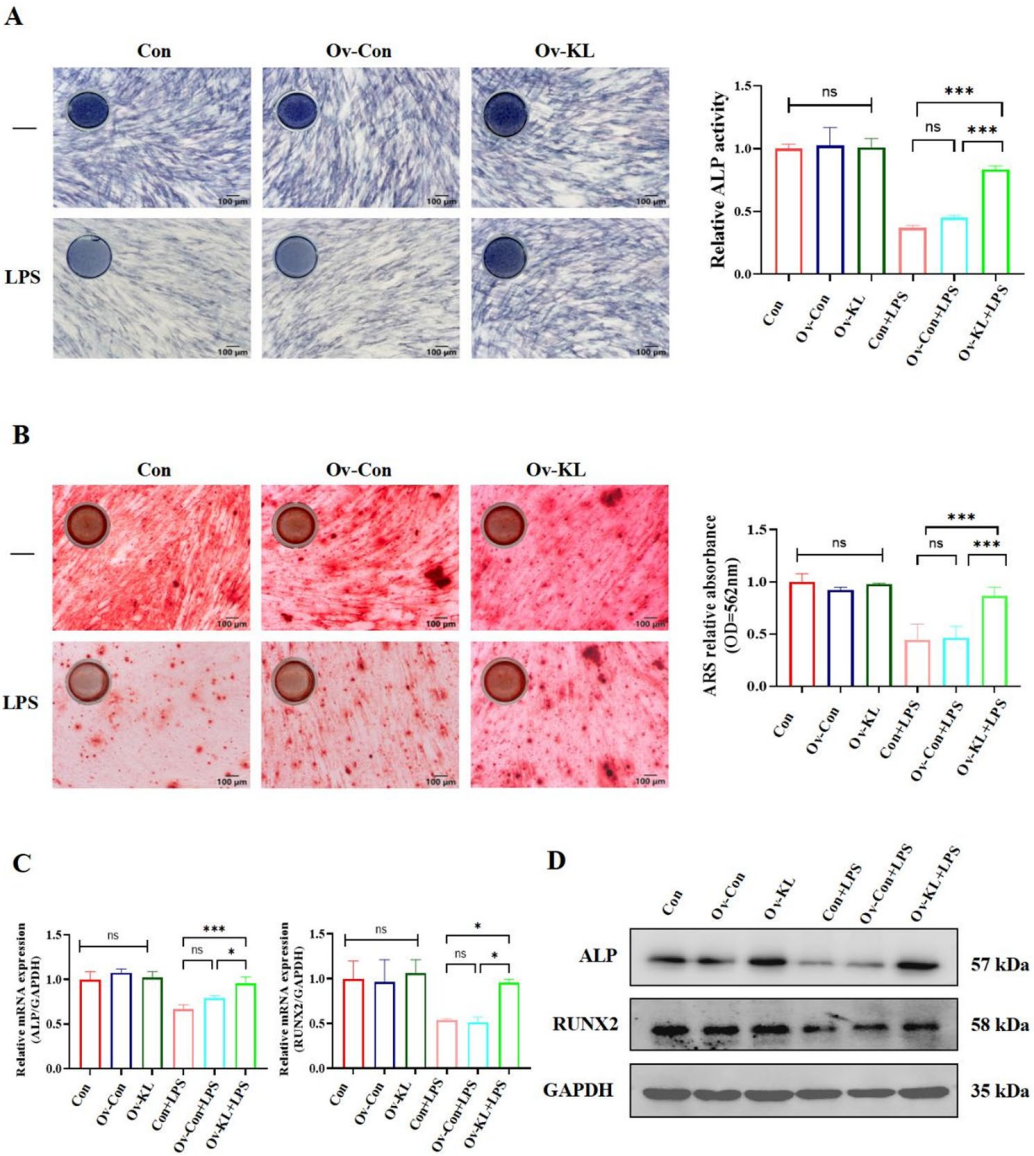
Experimental Assessment of Klotho overexpression on osteogenic function in hPDLSCs under the inflammatory environment. **A** ALP staining assay and quantification were performed (bar = 100 μm). **B** Mineralized nodule formation was observed and quantified using ARS (bar = 100 μm). **C** The mRNA expression levels of the ALP and RUNX2 by RT-qPCR. **D** The protein expression levels of the ALP and RUNX2 by Western blotting. Uncropped images were provided in Supplementary material 2: Original Images for Western blots. (*P < 0.05, ***P < 0.001, ns: not significant, *n* = 3)

Based on the findings that ferroptosis involved in inflammatory-mediated impairment of osteogenic function in hPDLSCs, we further investigated the impact of Klotho overexpression on osteogenesis under the inflammatory environment. ALP is a key marker of early osteogenesis. ALP staining and activity assays showed a substantial reduction in hPDSLC early osteogenic capacity under the inflammatory environment, while Klotho overexpression effectively preserved the osteogenic function of hPDLSCs under inflammation (Fig. 4A). Consistent with the ALP findings, the ARS experiment demonstrated similar protective effects (Fig. 4B). Moreover, Both RT-qPCR and Western blotting confirmed that Klotho overexpression maintained the expression levels of ALP and RUNX2 following inflammatory stimulation (Fig. 4C and D). These findings collectively demonstrated that Klotho serves as a crucial protector of osteogenic capacity in hPDLSCs under the inflammatory environment.

### hPDLSCs-ov-KL promotes periodontal tissue repair in mice with periodontitis

**Fig. 5:**
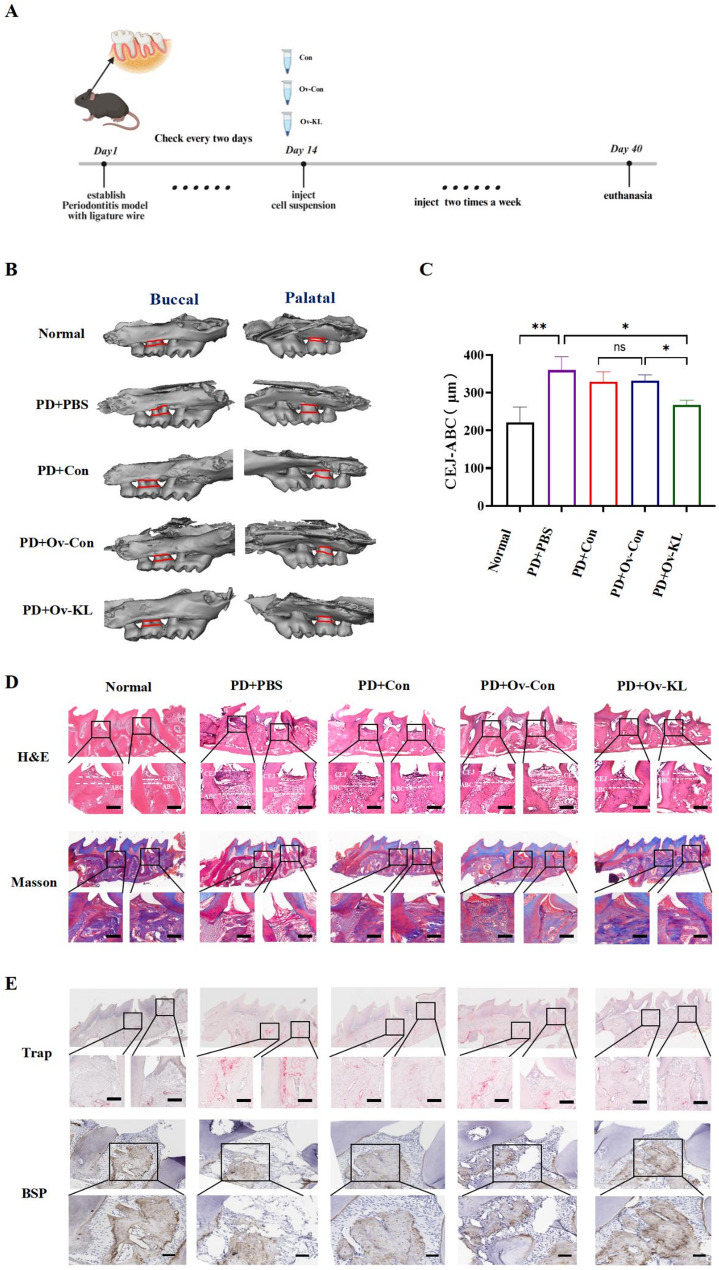
Regenerative effects of hPDLSCs-ov-KL treatment in periodontitis in mice. **A** The experimental design. Created in BioRender (agreement number: HY28QR7G1M). **B**, **C** Micro**-**CT images in each group. **D** H&E and Masson staining of the periodontal tissue (bar = 100 μm). **E** TRAP staining (bar = 100 μm) and Immunohistochemistry for BSP (bar = 200 μm) in the mice periodontal tissues. (*P < 0.05, **P < 0.01, ns: not significant, *n* = 6)

Prompted by the effects of Klotho overexpression on osteogenic function in vitro, we assessed the capacity of hPDLSCs-ov-KL for periodontal tissue regeneration in vivo. As outlined in Fig. 5A, experimental periodontitis was induced via ligature placement in mice, resulting in buccal and palatal alveolar bone resorption. Micro**-**CT analysis demonstrated attenuated bone resorption following local injections of hPDLSCs, hPDLSCs**-**ov**-**con, or hPDLSCs**-**ov**-**KL, with maximal efficacy in the induced + hPDLSCs**-**ov**-**KL group (Fig. 5B and C). H&E staining evaluated periodontal morphology, focusing on alveolar ridge height (ARH) and attachment levels. The induced group showed typical pathology: crestal bone loss and vertical ARH reduction. Compared to hPDLSCs and hPDLSCs**-**ov**-**con controls, the hPDLSCs**-**ov**-**KL group demonstrated significantly greater improvement in limiting crestal resorption and maintaining ridge integrity. Masson’s trichrome staining revealed reduced collagen fiber content in induced group, while hPDLSCs**-**ov**-**KL treatment counteracted degenerative collagen mostly (Fig. 5D). Additionally, Relative to other groups, hPDLSCs-ov-KL showed reduced osteoclasts and elevated bone sialoprotein (BSP) experssion levels (Fig. 5E). Taken together, hPDLSCs**-**ov**-**KL demonstrated superior efficacy over hPDLSCs and hPDLSCs-ov-con in treating periodontitis-induced bone loss in mice, with enhanced alveolar bone preservation, suppressed osteoclast formation, improved collagen reorganization and significant tissue repair.

### Klotho inhibited NOX4-driven ferroptosis to rescue the hPDLSC osteogenic function

**Fig. 6:**
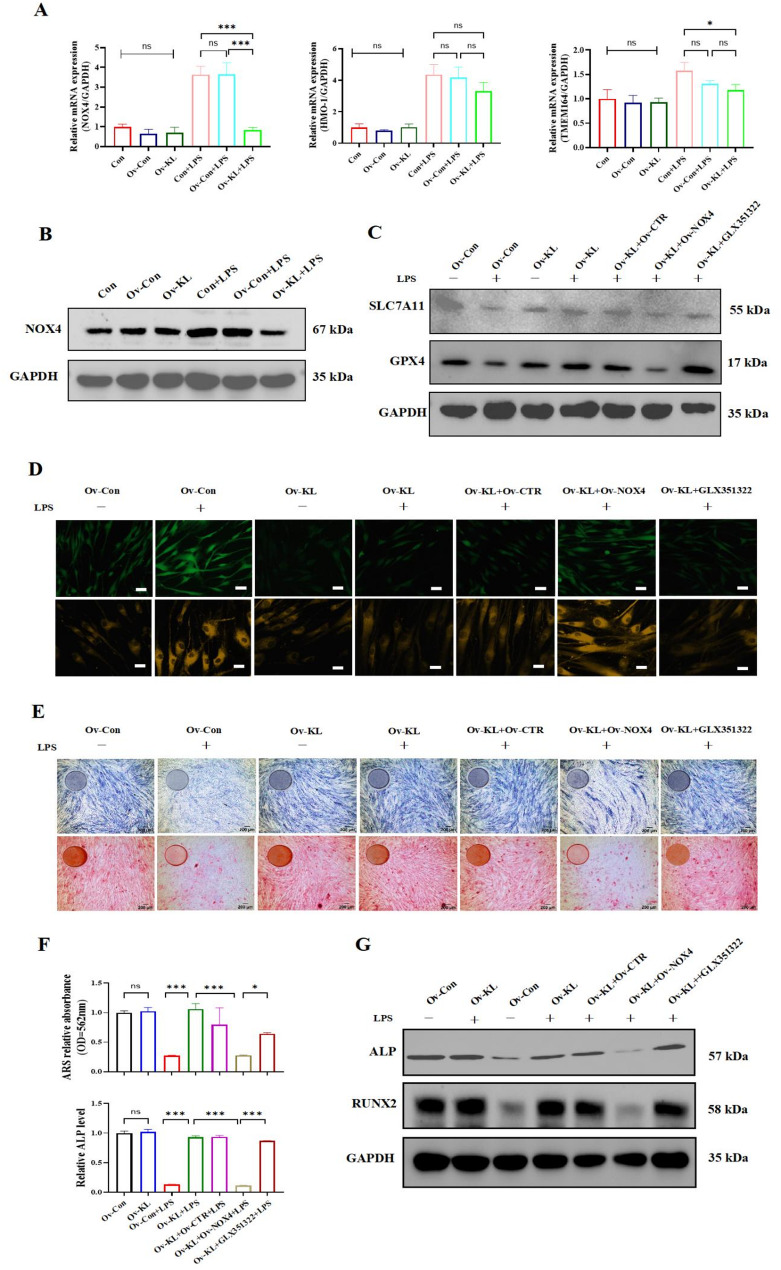
Impact of NOX4 on ferroptosis and osteogenic function in hPDLSCs-ov-KL under the inflammatory environment. **A** The mRNA expression levels of the *NOX4*,* HMO-1* and *TMEM164* genes in each group via RT-qPCR. **B** The protein expression levels of the NOX4 in each group via Western blotting. Uncropped images were provided in Supplementary material 2: Original Images for Western blots. **C** The results of Western blotting for the protein level of SLC7A11 and GPX4. Uncropped images were provided in Supplementary material 2: Original Images for Western blots. **D** Measurement of the ROS and intracellular Fe^2+^ levels (bar = 50 μm). **E** ALP staining assay and ARS were conducted (bar = 200 μm). **F** The quantitative results of ALP and ARS. **G** The protein expression levels of ALP and RUNX2 by Western blotting. Uncropped images were provided in Supplementary material 2: Original Images for Western blots. (*P < 0.05, ***P < 0.001, ns: not significant, *n* = 3)

In order to decipher how Klotho inhibited ferroptosis and maintained osteogenic function, we identified three upregulated genes related to ferroptosis, NOX4, HMOX1, TMEM164, via an expression bubble diagram (Fig. S1F). Subsequently, we evaluated the expression levels of the three genes between hPDLSCs-ov-KL and control groups. NOX4 exhibited the most significant alteration under the inflammatory environment, and the other two genes showed non-significant changes (Fig. 6A). Consistently, Western blotting confirmed that Klotho overexpression significantly attenuated the NOX4 upregulation under the inflammatory environment (Fig. 6B). To validate the pivotal role of NOX4 in Klotho-mediated regulation in hPDLSCs, we performed functional experiments combining NOX4 overexpression and NOX4 inhibitor. We employed a NOX4 overexpression plasmid for NOX4 overexpression (Fig. S4) and the NOX4 inhibitor — GLX351322 for NOX4 inhibition and comprehensive ferroptosis assays and osteogenic functional analyses were performed. The result revealed that in the inflammatory environment, NOX4 overexpression via plasmid transfection in hPDLSCs-ov-KL significantly downregulated the protein level of anti-ferroptosis genes *GPX4* and *SLC7A11*, with increased intracellular ROS and Fe^2+^ level, leading to compromised Klotho function. Meanwhile, the inhibition of NOX4 presented to be potentiated Klotho function (Fig. 6C and D). At the same time, NOX4 overexpression abolished protective effects of Klotho on hPDLSC osteogenic function under the inflammatory environment (Fig. 6E, F and G). In summary, these findings identified NOX4 as a critical molecule through which Klotho governs both ferroptosis resistance and osteogenic protection in hPDLSCs.

## Discussion

In the periodontitis microenvironment, the inflammatory stimuli suppressed the functional properties of hPDLSCs, leading the failure of periodontal tissue regeneration [33, 34]. Ferroptosis has emerged to play a crucial role in various inflammatory diseases [35]. In periodontitis, the inflammatory microenvironment drived ferritin accumulation and elevated lipid peroxidation in resident periodontal cells, which amplified the inflammatory response within tissues, thereby exacerbating periodontitis severity [36]. Within diseased periodontium, the persistent upregulation of ROS collectively created conditions contributing to ferroptosis in hPDLSCs [37]. However, the precise mechanisms how ferroptosis involved in the hPDLSC function and periodontitis progression remains unclear. Klotho, the anti-aging protein was known for its antioxidant properties that protect against oxidative stress in various tissues. Our previous research showed that Klotho reduced ROS and preserved the osteogenic function of hPDLSCs under oxidative stress [18]. In this research, bioinformatic analysis and validation experiment identified that inflammation in hPDLSCs caused impaired osteogenic function dominated by increased ferroptosis level and paralleled by Klotho downregulation. This reveals Klotho as a crucial mediator coupling ferroptosis signaling to hPDLSC dysfunction.

Notably, our data implied that Klotho overexpression could counteract increased ferroptosis and ROS levels in hPDLSCs under the inflammatory environment. This aligns with emerging evidence that connected inflammatory signaling to ferroptosis via iron dysregulation and redox imbalance [35]. Pro-inflammatory cytokines such as IL-6 and TNF-α may exacerbate iron overload by modulating ferritin synthesis, leading the exacerbation of tissue inflammation [38]. The Klotho protein, when localized to the plasma membrane (mKlotho), can function as a coreceptor for FGF23, regulating mineral metabolism. Concurrently, proteolytic cleavage releases soluble Klotho (sKlotho) that exert pleiotropic effects, modulating Wnt signaling and ion channel activity, which are crucial for cellular homeostasis and stress resistance [39, 40]. The protective effects observed in our study may involve both forms of Klotho. Researches have shown that the efficacy of Klotho may result from its direct interactions with cell surface receptors, enabling more effective regulation in iron homeostasis and inflammatory interplay in neurological disorders [41]. Herein, Klotho has shown to be a promising molecule that links ferroptosis, oxidative stress and inflammation, offering potential for sustaining hPDLSC biogenesis in the inflammatory environment.

Further, the effects that Klotho sustained hPDLSC osteogenic potential under inflammatory stimuli were explored in this study, showing Klotho effects on preserving osteogenic capacity coincided with the inhibition of lipid peroxidation and ROS level. These results align with the recent studies that Klotho function has been explored from an anti-aging protein associated with aging-related pathologies to a regulator of inflammation-related diseases across multiple organ systems [42, 43]. The pleiotropic nature of Klotho extends to encompass crucial roles in skeletal homeostasis. Spatial-temporal expression analyses show the presence of Klotho across osteogenic lineage cells and its regulatory involvement in bone remodeling cascades [44–47]. Alveolar bone repair models show that Klotho deficiency impairs osteogenic function of oral mesenchymal progenitors under the inflammatory condition which can be rescued via Klotho-mediated suppression of osteoclastgenesis [48]. Our results extend this paradigm by showing that endogenous Klotho overexpression preserves the osteogenic potential of the hPDLSCs especially under the inflammatory environment and in a mouse model of periodontitis, as evidenced by the coordinated evaluations of ALP activity, mineralized nodule formation, and alveolar bone regeneration.

As for the molecular mechanism, we demonstrated that Klotho overexpression inhibits NOX4 expression to reduce lipid peroxidation and ferroptosis level. This aligns with previous studies identifying NOX4-mediated ROS production as a crucial player in initiating ferroptosis and linking oxidative stress to cellular dysfunction in inflammatory scenarios [25]. As a key ROS source, NOX4 critically regulates bone metabolism. NOX4 deficiency elevates bone mass and decreases osteoclast activity [49, 50], disrupting bone homeostasis by stimulating osteoclastogenesis [51]. Our findings and results of other researches collectively reveal NOX4 as a master regulator of bone pathophysiology. In our study, we contributed to the growing understanding that NOX4 plays a crucial role in enhancing hPDLSC ferroptosis under the inflammatory environment and Klotho exerts a protective mechanism against ferroptosis by targeting NOX4, opening new avenues for therapeutic interventions in inflammatory bone diseases.

Recently, ferroptosis-related mechanisms in periodontitis have been found to link to age-associated systemic diseases such as Alzheimer’s disease, chronic obstructive pulmonary disease, and diabetes [52]. Under the pathophysiological context of inflammaging, the dysfunction of stem cells under inflammation fuels age-associated degenerative processes [53]. Our results suggest that Klotho can substantially mitigate ferroptosis and preserve the osteogenic function of hPDLSCs in the inflammatory environment. This anti-inflammaging mechanism may explain the superior stemness preservation of hPDLSCs to conventional anti-inflammatory strategies.

Our study highlighted the function and mechanism of Klotho in preserving hPDLSC osteogenic function under the inflammatory environment. While this study highlights the overall therapeutic potential of Klotho, the distinct contributions of its different forms remain to be fully elucidated. Future investigations should aim to dissect the specific mechanisms by which each Klotho isoform influences hPDLSC function. Future studies should consider using spatial multi-omics sequencing of clinical periodontal samples at single-cell resolution, therefore decoding Klotho effects and mechanisms associated to immune cell modulating and tissue remodeling in the whole microenvironment, ultimately informing targeted therapies for periodontitis and other inflammatory bone diseases.

## Conclusion

Our study highlights the significant protective effect of Klotho on counteracting hPDLSC ferroptosis via the inhibition of NOX4 expression, therefore restoring the impaired osteogenic function of hPDLSCs in both in vitro inflammatory environment and in vivo periodontitis animal model. Thus, our study underscores the crucial role of Klotho in protecting the hPDLSC osteogenic function against the detrimental effects of ferroptosis in inflammatory environment, which presents a targeted therapies for hPDLSC application in periodontal tissue regeneration and inflammatory bone diseases.

## Supplementary Information

Supplementary Material 1.

Supplementary Material 2.

Supplementary Material 3.

## Data Availability

No datasets were generated or analysed during the current study.

## Abbreviations

H&E: Hematoxylin and eosin
hPDLSCs: Human periodontal ligament stem cells
Micro-CT: Micro-computed Tomography Examination
MSC: Mesenchymal stem cell
NOX4: NADPH oxidase 4
NC: Nitrocellulose
UMAP: Uniform manifold approximation and projection
α-MEM: α-minimum essential medium
ROS: Reactive oxygen species
LPS: Lipopolysaccharide
ALP: Alkaline phosphatase
ARS: Alizarin red staining
MDA: Malondialdehyde
BSP: Bone sialoprotein
PCR: Polymerase chain reaction
RT-qPCR: Quantitative reverse transcription polymerase chain reaction
SDS-PAGE: Sodium dodecyl sulfate-polyacrylamide gel
TEM: Transmission electron microscopy
