# Supplementary material for: Klotho protects the osteogenic function of human periodontal ligament stem cells in periodontitis by inhibiting NOX4-mediated ferroptosis
Source: Stem Cell Res Ther. 2026 Jan 16;17:81. doi: 10.1186/s13287-026-04894-w (PMC12892636; doi:10.1186/s13287-026-04894-w)
Supplement: Supplementary file 2 — Supplementary Material 2. [file 13287_2026_4894_MOESM2_ESM.pdf]

Original Images for Western blots

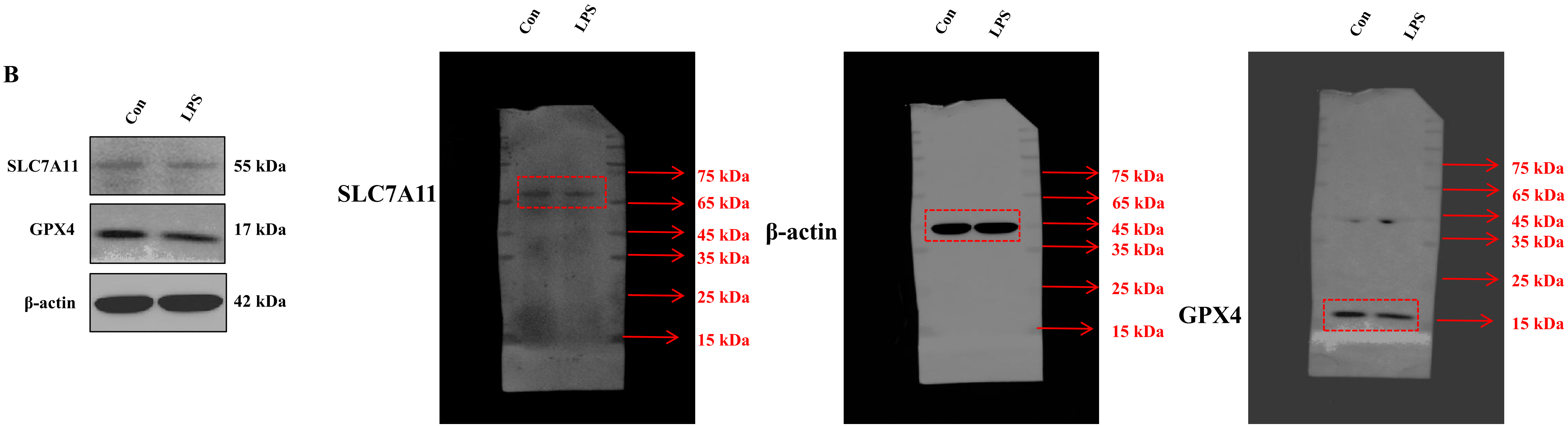

Uncropped Western blot images corresponding to Fig. 2B

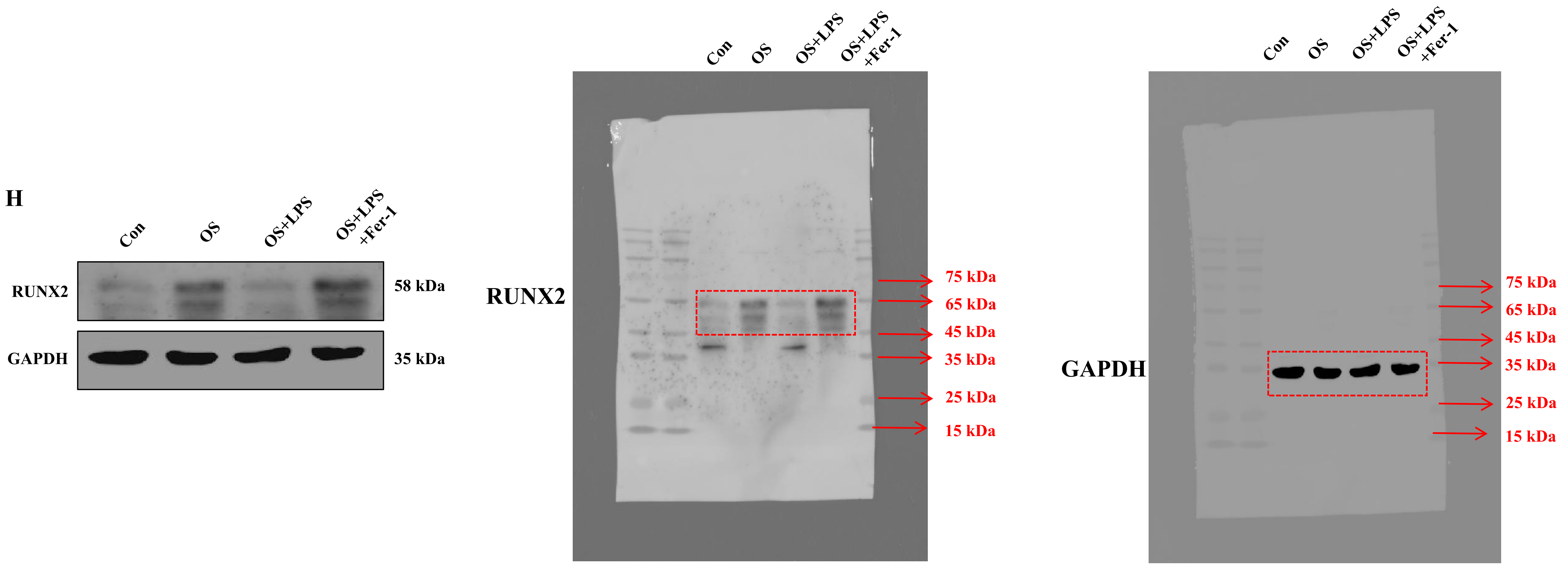

Uncropped Western blot images corresponding to Fig. 2H

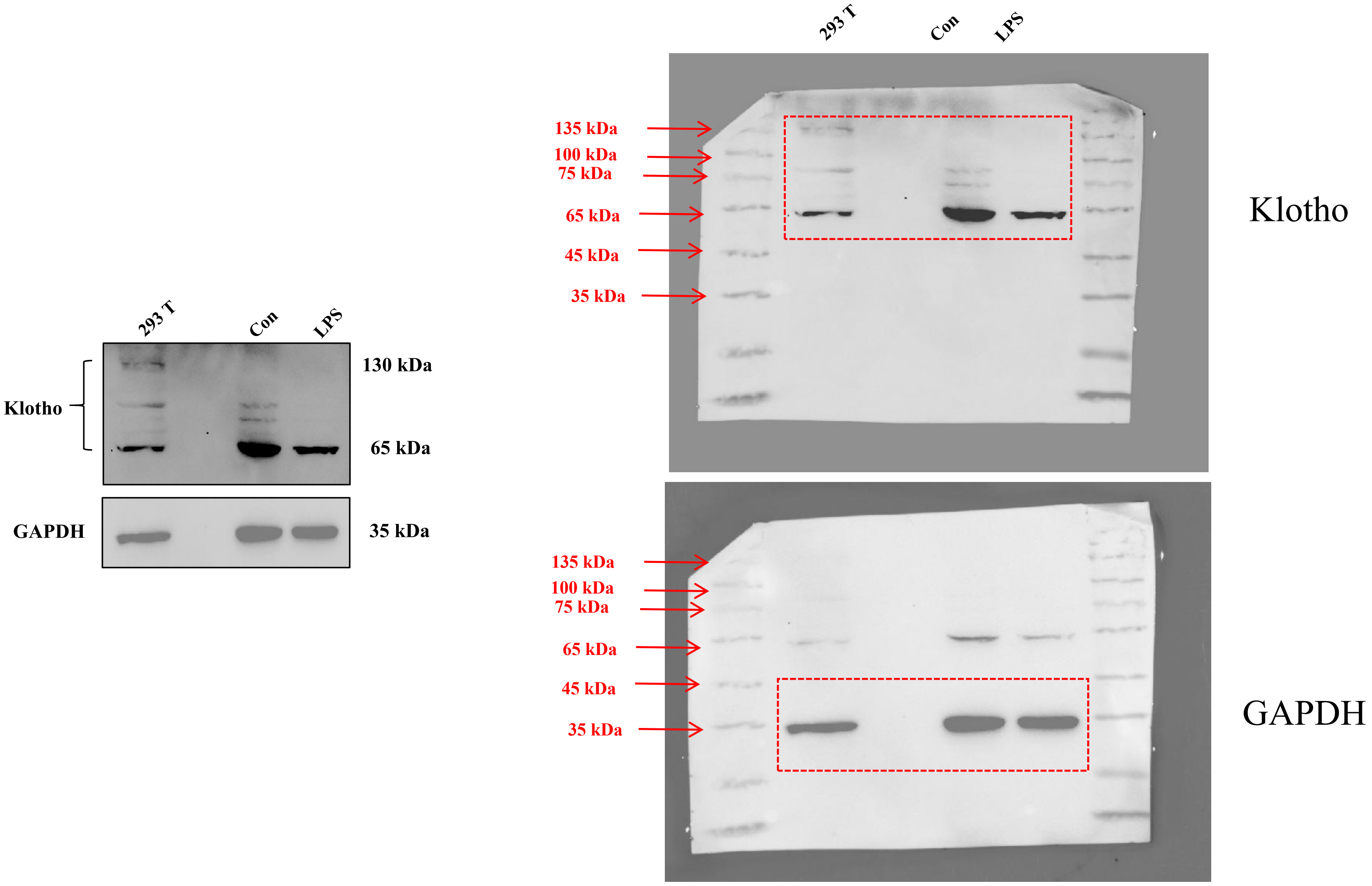

Uncropped Western blot images corresponding to Fig. 3A

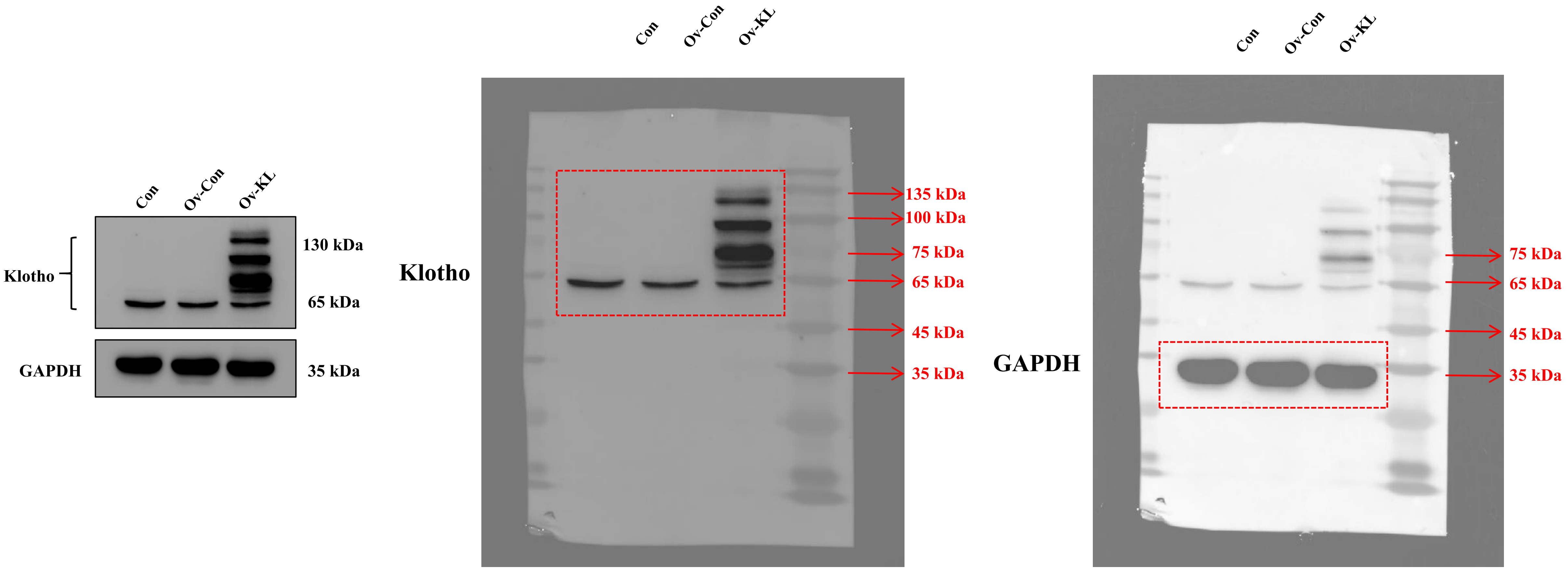

Uncropped Western blot images corresponding to Fig. 3B

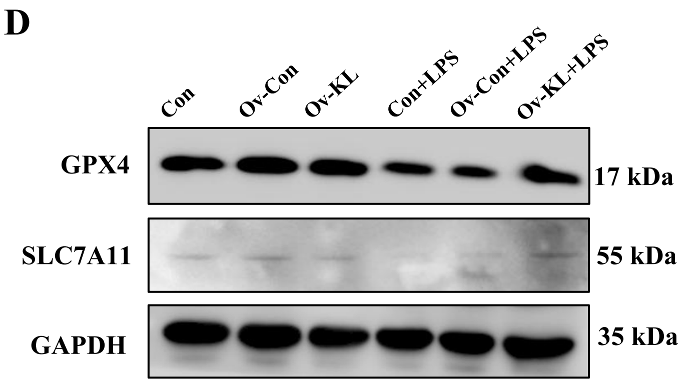

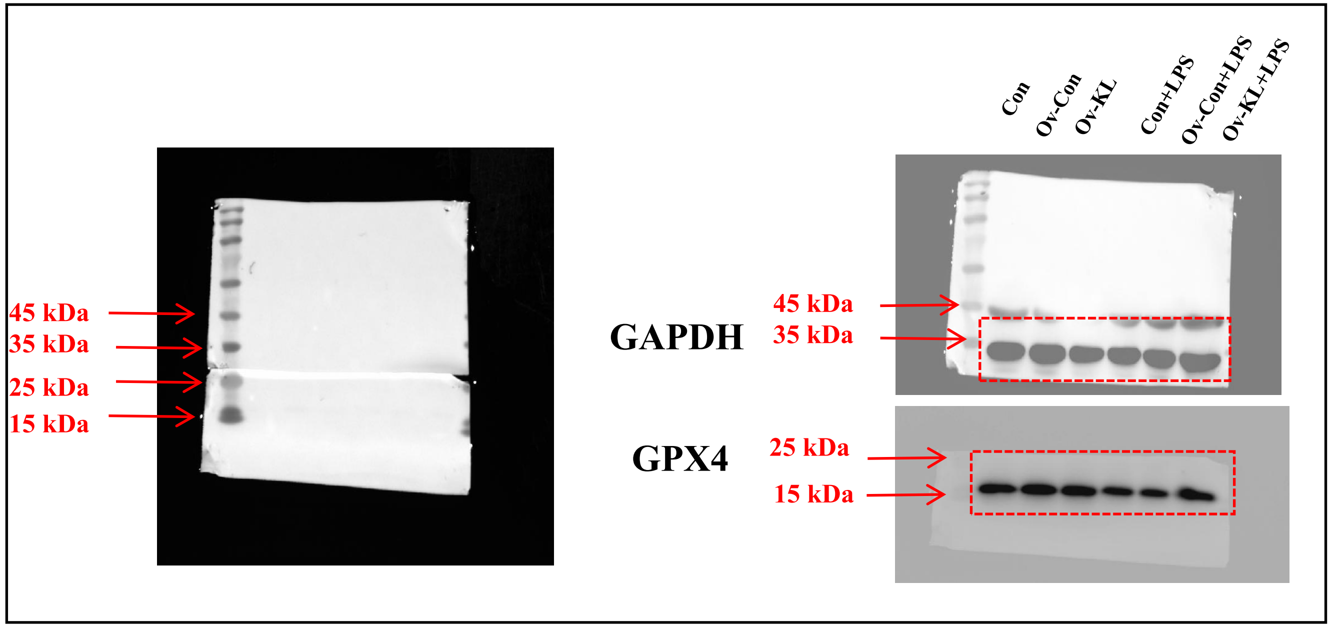

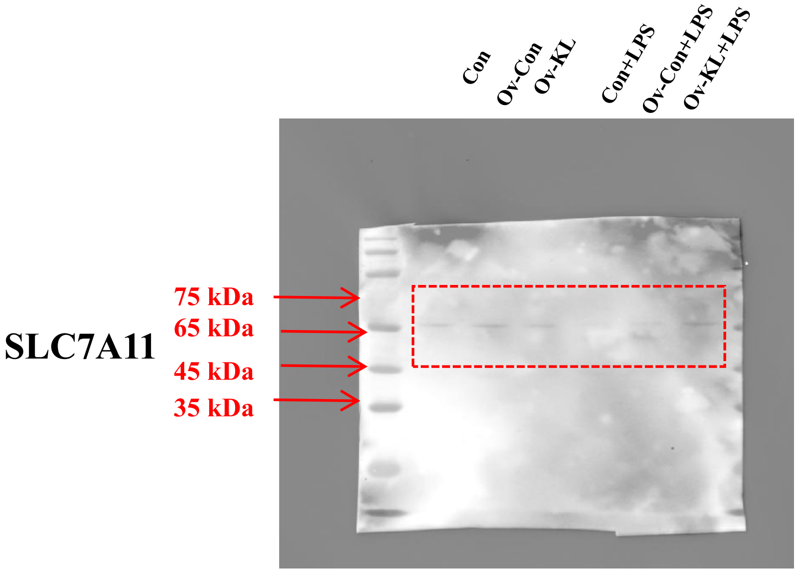

Uncropped Western blot images corresponding to Fig. 3D

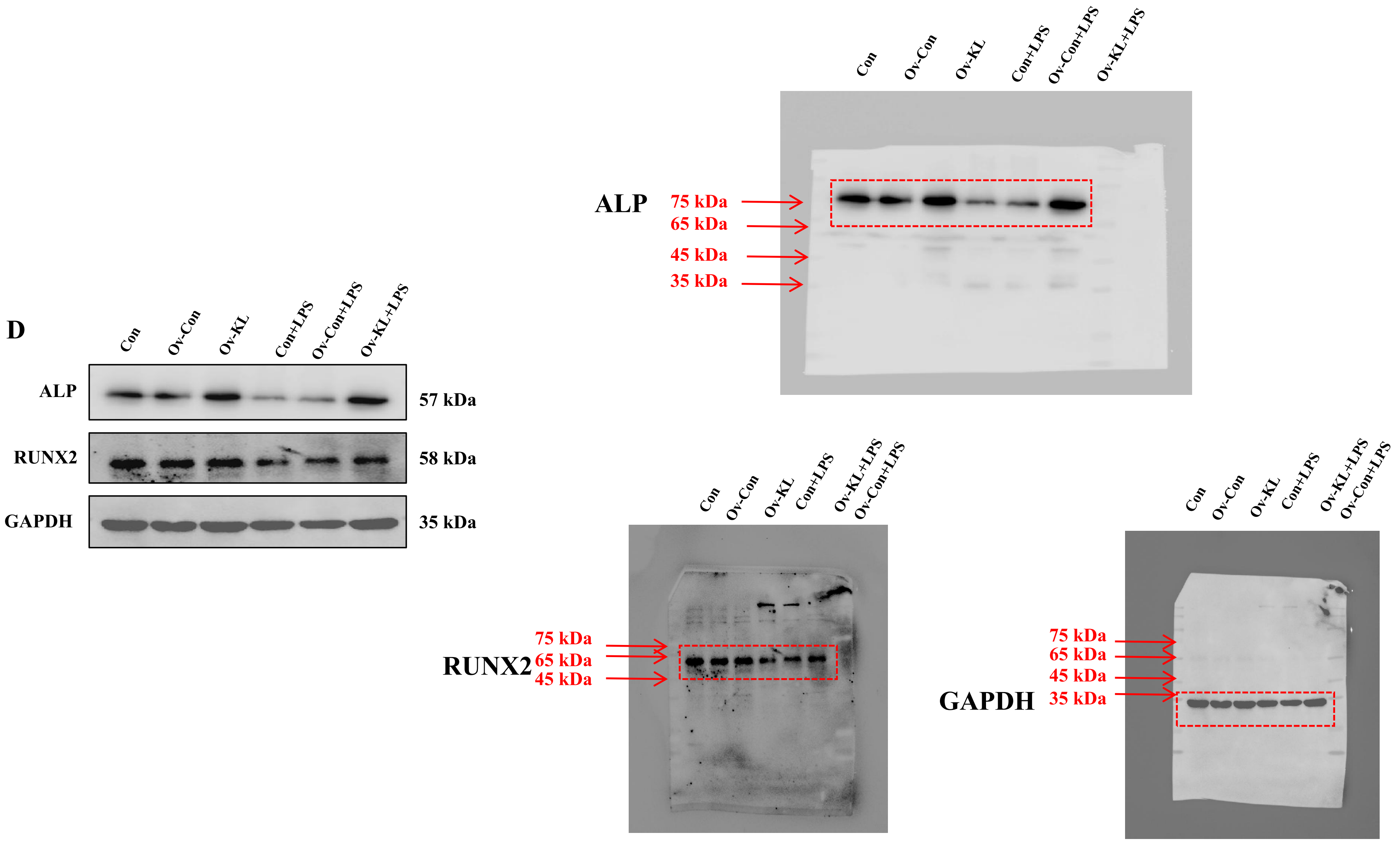

Uncropped Western blot images corresponding to Fig. 4D

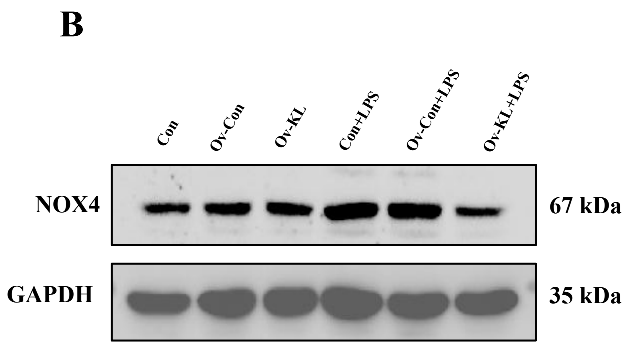

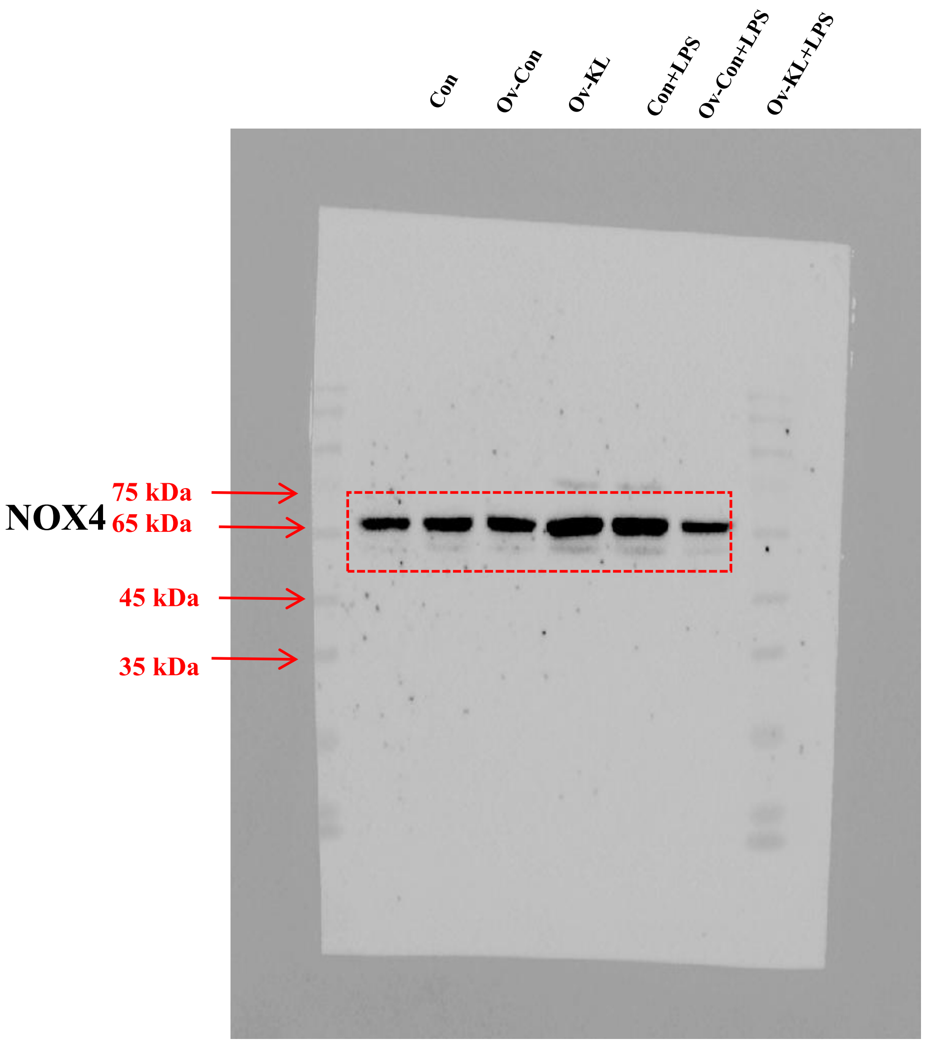

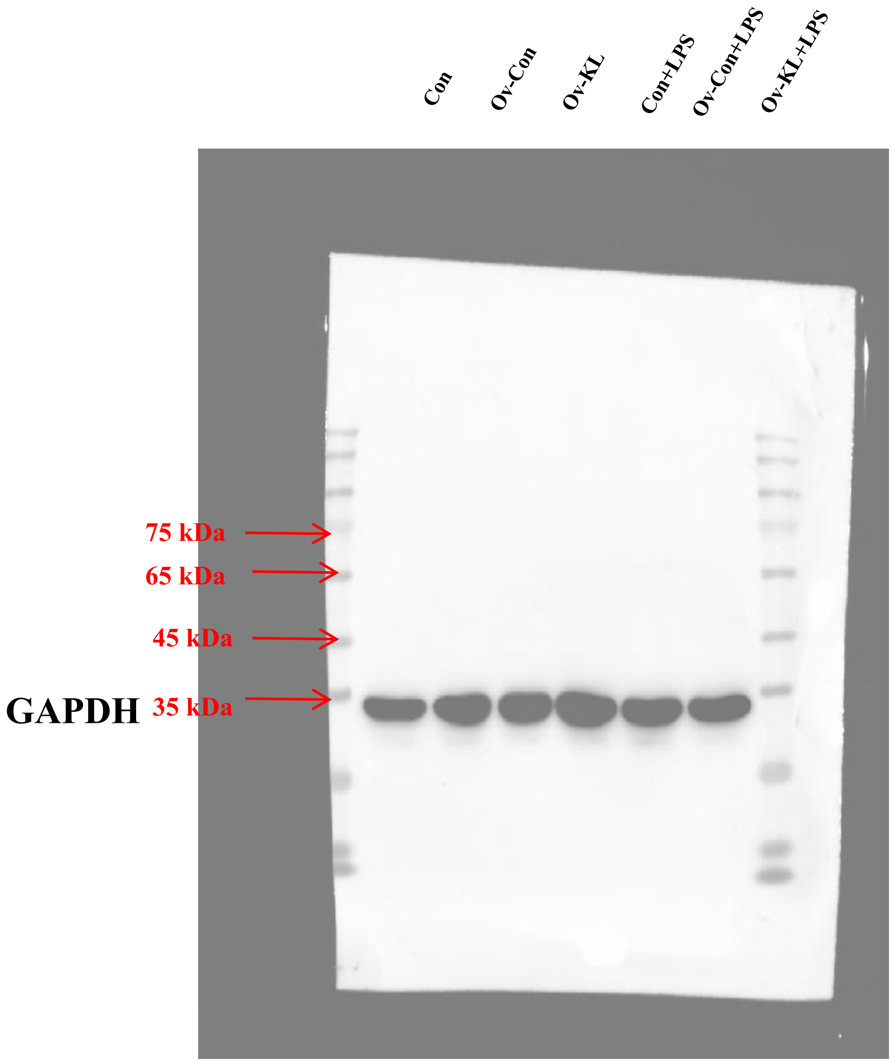

Uncropped Western blot images corresponding to Fig. 6B

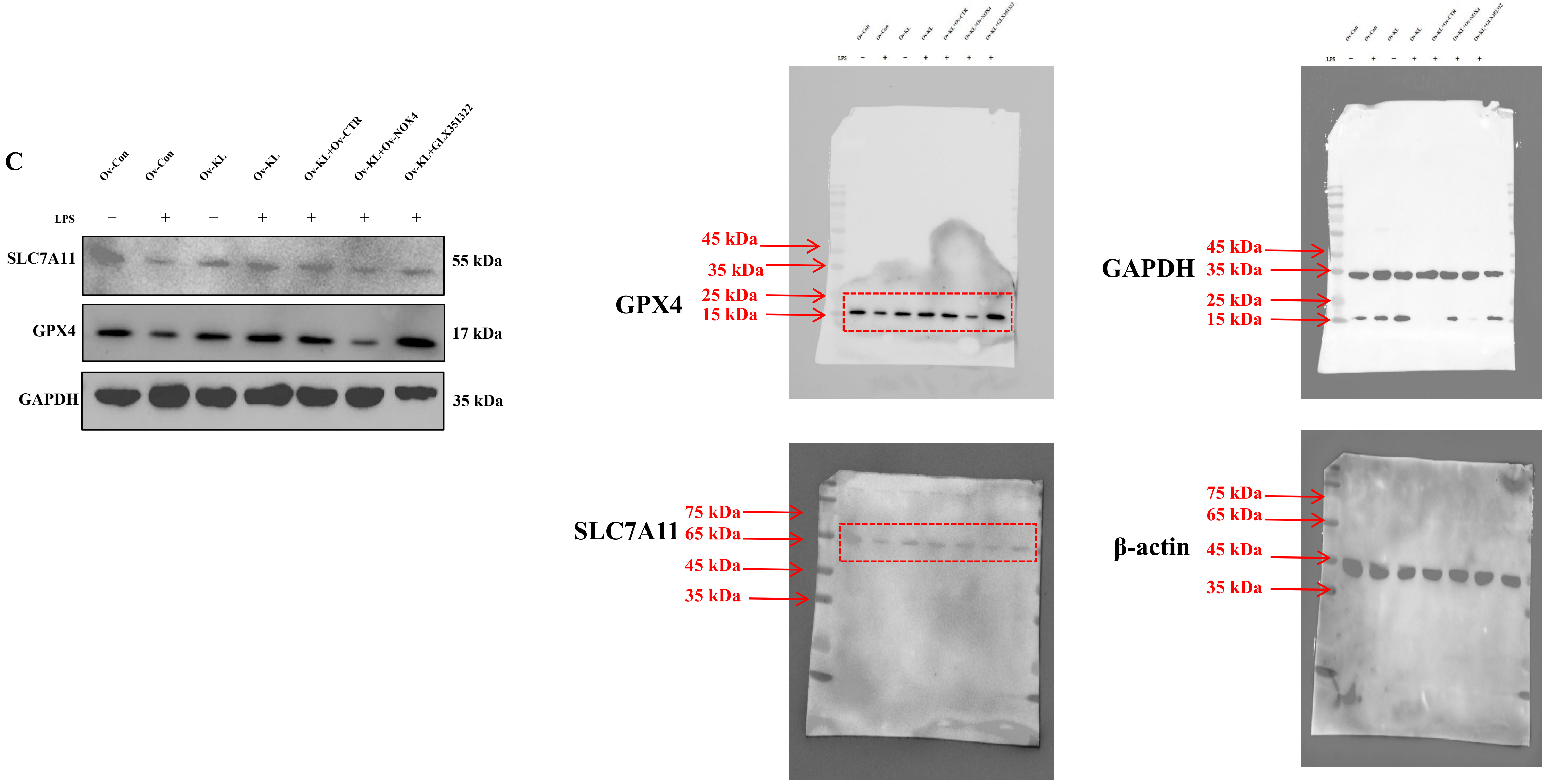

Uncropped Western blot images corresponding to Fig. 6C

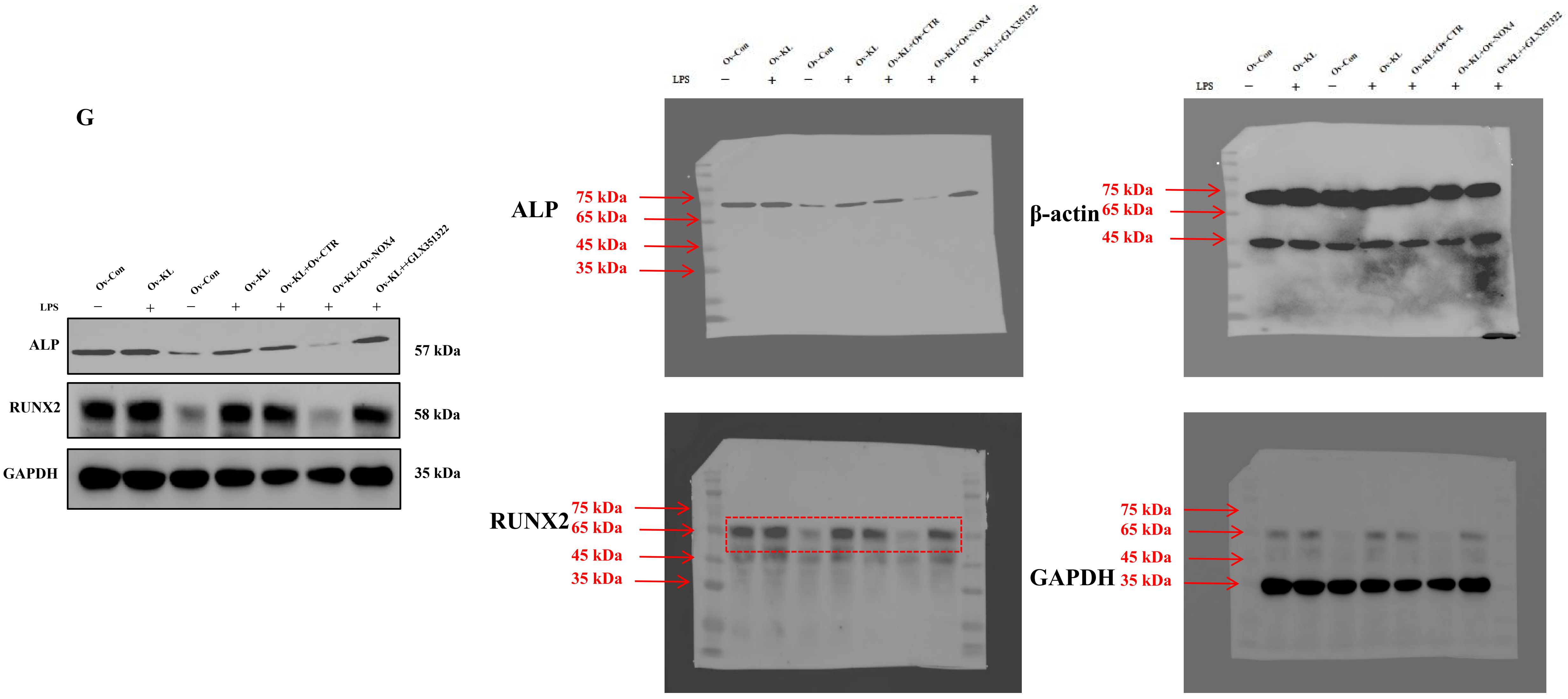

Uncropped Western blot images corresponding to Fig. 6G

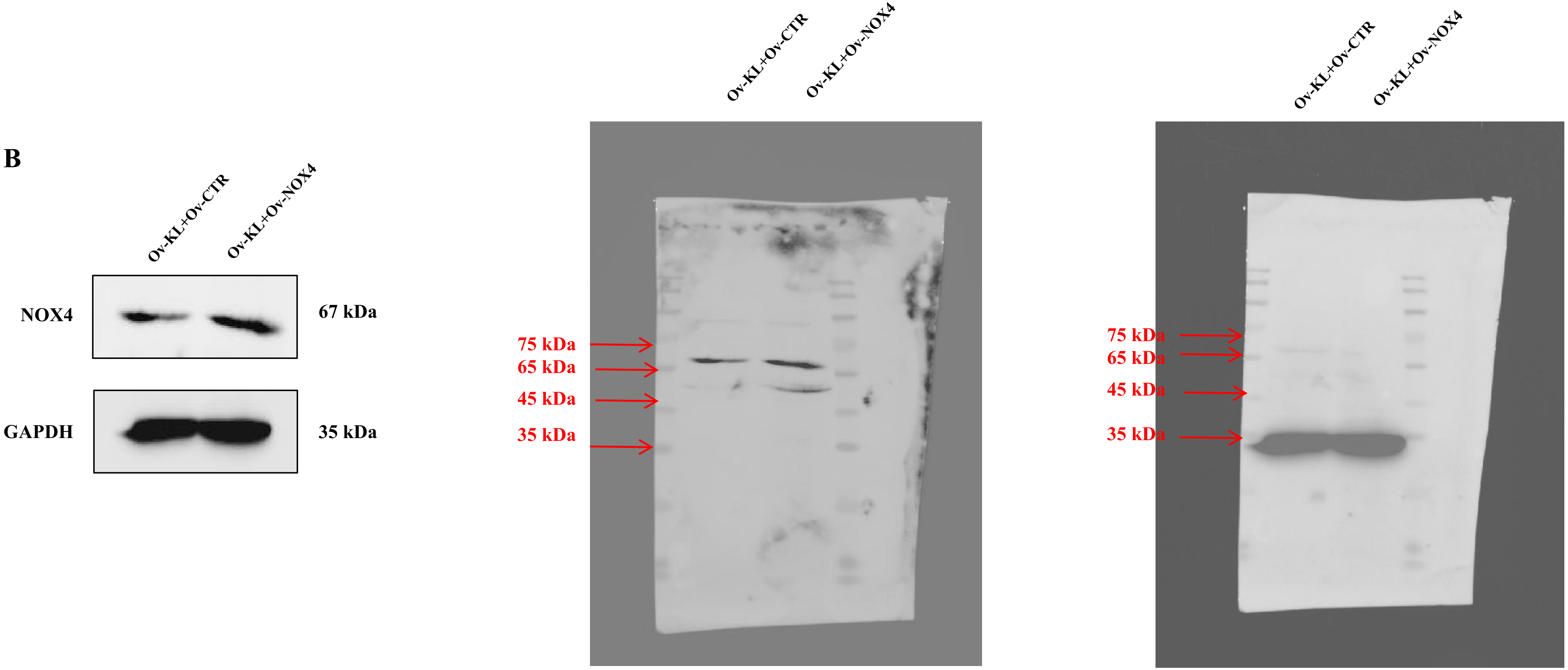

Uncropped Western blot images corresponding to Fig. S4B
