# Supplementary material for: Klotho protects the osteogenic function of human periodontal ligament stem cells in periodontitis by inhibiting NOX4-mediated ferroptosis
Source: Stem Cell Res Ther. 2026 Jan 16;17:81. doi: 10.1186/s13287-026-04894-w (PMC12892636; doi:10.1186/s13287-026-04894-w)
Supplement: Supplementary file 1 — Supplementary Material 1. [file 13287_2026_4894_MOESM1_ESM.docx]

**Supplementary material 1**

Table S1 Sequences of RT-qPCR primers

| Species | Gene | Sequence |
| --- | --- | --- |
| Human | *Klotho* | F: 5′- CTCGGGGTCACTCACTACC -3′ |
|  |  | R: 5′- CTCCGCGTAATCCCTGAAGT-3′ |
|  | *Gpx4* | F: 5′- GAGGCAAGACCGAAGTAAACTAC -3′ |
|  |  | R: 5′- CCGAACTGGTTACACGGGAA -3′ |
|  | *Slc7a11* | F: 5′- TCTCCAAAGGAGGTTACCTGC -3′ |
|  |  | R: 5′- AGACTCCCCTCAGTAAAGTGAC -3′ |
|  | *Alp* | F: 5′- AGCACTCCCACTTCATCTGGAA -3′ |
|  |  | R: 5′- GAGACCCAATAGGTAGTCCACATTG -3′ |
|  | *Runx2* | F: 5′- TGGTTACTGTCATGGCGGGTA -3′  R: 5′- TCTCAGATCGTTGAACCTTGCTA -3′ |
|  | *Nox4* | F: 5′- TGTGCCGAACACTCTTGGC -3′  R: 5′- ACATGCACGCCTGAGAAAATA -3′ |
|  | *Gapdh* | F: 5′- GGAGCGAGATCCCTCCAAAAT -3′ |
|  |  | R: 5′- GGCTGTTGTCATACTTCTCATGG -3′ |

**Fig S1.** Single-cell RNA sequencing showed ferroptosis pathway activation in mesenchymal stem cells (MSCs) from patients with periodontitis. (**A-C**) UMAP plots revealed periodontal ligament cell subpopulations in four healthy patients and five patients with periodontitis. (**D**) Bubble plot illustrated markers of cell subpopulations. (**E**) Box plot revealed the differentiation potential of various mesenchymal cell subpopulations. (**F**) Bubble plot revealed the expression of ferroptosis-related genes in the MSCs.

**Fig S2.** Isolation and characterization of the hPDLSCs. **(A,B)** The cell morphology of primary hPDLSCs and hPDLSCs following passage (bar = 200 µm). **(C)** Colony formation ability of the hPDLSCs (bar = 50 µm). **(D)** Alizarin red (bar = 500 µm), Oil red O (bar = 50 µm), and Alcian blue staining (bar = 400 µm) of the hPDLSCs after osteogenic, adipogenic, or chondrogenic induction. **(E)** Tissue origin identification of hPDLSCs (bar = 50 µm). **(F)** Surface markers identification of hPDLSCs via flow cytometry analysis.

**Fig S3.** Establish an *in vitro* inflammation model of hPDLSCs. **(A)** IncuCyte proliferation graphs of hPDLSCs in gradient concentrations. **(B)** ARS was utilized to confirm that the 4 μg/ mL LPS effectively impairs osteogenesis (bar = 100 µm). (**P* < 0.05, ****P* < 0.001, ns: not significant, n = 3)

**Fig S4.** Verification of transient overexpression of NOX4 in Ov-KL. **(A)** The mRNA expression level of the *NOX4* gene using RT-qPCR. **(B)** The protein expression level of NOX4 observed via Western blotting. Uncropped images were provided in Supplementary material 2: Original Images for Western blots. (****P* < 0.001, n=3)
